# Sequential Ubiquitination and Phosphorylation Epigenetics Reshaping by MG132‐Loaded Fe‐MOF Disarms Treatment Resistance to Repulse Metastatic Colorectal Cancer

**DOI:** 10.1002/advs.202301638

**Published:** 2023-06-11

**Authors:** Zhaoting Bu, Jianjun Yang, Yan Zhang, Tao Luo, Chao Fang, Xiayi Liang, Qiuxia Peng, Duo Wang, Ningjing Lin, Kun Zhang, Weizhong Tang

**Affiliations:** ^1^ Department of Gastrointestinal Surgery Guangxi Medical University Cancer Hospital Guangxi Medical University. No. 71 Hedi Road Nanning Guangxi 530021 P. R. China; ^2^ Central Laboratory and Department of Orthopaedics Shanghai Tenth People's Hospital Tongji University School of Medicine Tongji University. No. 301 Yan‐chang‐zhong Road Shanghai 200072 P. R. China; ^3^ Central Laboratory Sichuan Academy of Medical Sciences Sichuan Provincial People's Hospital University of Electronic Science and Technology of China No. 32, West Second Section, First Ring Road Chengdu Sichuan 610072 P. R. China

**Keywords:** chemodynamics‐ignited oxidative stress, epigenetics reshaping, metastatic colorectal cancer, sequential ubiquitination and phosphorylation modulations, tumor microenvironment

## Abstract

Abnormal epigenetic regulation is identified to correlate with cancer progression and renders tumor refractory and resistant to reactive oxygen species (ROS)‐based anti‐tumor actions. To address it, a sequential ubiquitination and phosphorylation epigenetics modulation strategy is developed and exemplified by the well‐established Fe‐metal‐organic framework (Fe‐MOF)‐based chemodynamic therapy (CDT) nanoplatforms that load the 26S proteasome inhibitor (i.e., MG132). The encapsulated MG132 can blockade 26S proteasome, terminate ubiquitination, and further inhibit transcription factor phosphorylation (e.g., NF‐*κ*B p65), which can boost pro‐apoptotic or misfolded protein accumulations, disrupt tumor homeostasis, and down‐regulate driving genes expression of metastatic colorectal cancer (mCRC). Contributed by them, Fe‐MOF‐unlocked CDT is magnified to considerably elevate ROS content for repulsing mCRC, especially after combining with macrophage membrane coating‐enabled tropism accumulation. Systematic experiments reveal the mechanism and signaling pathway of such a sequential ubiquitination and phosphorylation epigenetics modulation and explain how it could blockade ubiquitination and phosphorylation to liberate the therapy resistance to ROS and activate NF‐*κ*B‐related acute immune responses. This unprecedented sequential epigenetics modulation lays a solid foundation to magnify oxidative stress and can serve as a general method to enhance other ROS‐based anti‐tumor methods.

## Graphical abstract

A sequential ubiquitination and phosphorylation epigenetics modulation strategy is established to enhance chemodynamic therapy (CDT) against colorectal cancer (CRC), wherein a Fe‐metal‐organic framework‐based CDT nanoplatform is used to load the 26S proteasome inhibitor. This strategy could boost pro‐apoptotic or misfolded protein accumulations, disrupt tumor homeostasis, promote apoptosis via enriching reactive oxygen species, and down‐regulate driving gene expression of CRC.

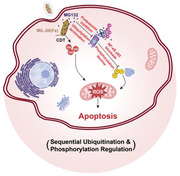

## 1 Introduction

The transcriptional epigenetic modifications that include intracellular DNA methylation, covalent modification of histones (e.g., acetylation, methylation, ubiquitination, and phosphorylation), and chromatin rearrangement have been accepted to be implicated in many biological activities.^[^ ^1^, ^2^ ^]^ In particular, abnormal epigenetic regulation is identified to correlate with the early‐stage and progression of cancer,^[^ ^3^, ^4^ ^]^ which also renders the tumor refractory and resistant to routine treatment methods.^[^ ^5^, ^6^ ^]^ Typically, the ubiquitin‐proteasome system (UPS) can facilitate pro‐apoptotic protein degradation and anti‐apoptotic accumulation, and perform like autophagy to protect cancer cells from damages or apoptosis to reactive oxygen species (ROS)‐based anti‐tumor treatment via inducing robust resistances.^[^ ^7^, ^8^, ^9^, ^10^ ^]^ Currently, there are many ROS‐based anti‐tumor treatment methods,^[^ ^11^, ^12^ ^]^ typically such as sonodynamic therapy (SDT), chemodynamic therapy (CDT), photodynamic therapy (PDT), and radiotherapy.^[^ ^13^, ^14^, ^15^, ^16^, ^17^ ^]^ Nevertheless, these methods are confronted with poor treatment efficiency and disappointed clinical translation due to the complex tumor microenvironment (TME) even though structure design elevates the production efficiency of ROS.^[^ ^18^, ^19^, ^20^, ^21^ ^]^ Despite achieving numerous inspiring achievements in TME modulation using nanomedicine after rational design,^[^ ^22^, ^23^ ^]^ only several TME types were modulated, for example, hypoxia, immune, inflammation, and redox balance.^[^ ^24^, ^25^, ^26^, ^27^, ^28^, ^29^, ^30^, ^31^ ^]^ Disappointingly, epigenetics modulation that directly dictates tumor progression, evolution, and metastasis still receives little attention,^[^ ^32^ ^]^ and even rare attempts to enhance the currently‐prevalent ROS‐based anti‐tumor efficiency have been made yet.

In this report, we constructed a metal‐organic framework (MOF) (i.e., NH_2_‐MIL‐88)‐based CDT nanoplatform to reach the sequential ubiquitination and phosphorylation epigenetics regulations after loading proteasome inhibitors (MG132) (MIL‐88‐MG132), which was leveraged to boost CDT‐ignited ROS level and promote CDT efficiency against metastatic colorectal cancer (mCRC). Depending on the numerous merits, including robust structural stability, tunable physio‐chemical properties, and no additional CDT sensitizers, MOF has been identified as an excellent drug carrier and CDT executor after using Fe, Cu, or Mn as active centers.^[^ ^33^ ^]^ Herein, NH_2_‐MIL‐88(Fe) MOF featuring high Fenton reaction activity that was usually applied in environmental catalysis was used to enable CDT against mCRC,^[^ ^34^, ^35^ ^]^ because Fe active centers in MIL‐88(Fe) can respond to intratumoral H_2_O_2_ to give birth to excessive hydroxyl radicals (∙OH) and bring about imbalanced oxidative stress.

**Figure 1:**
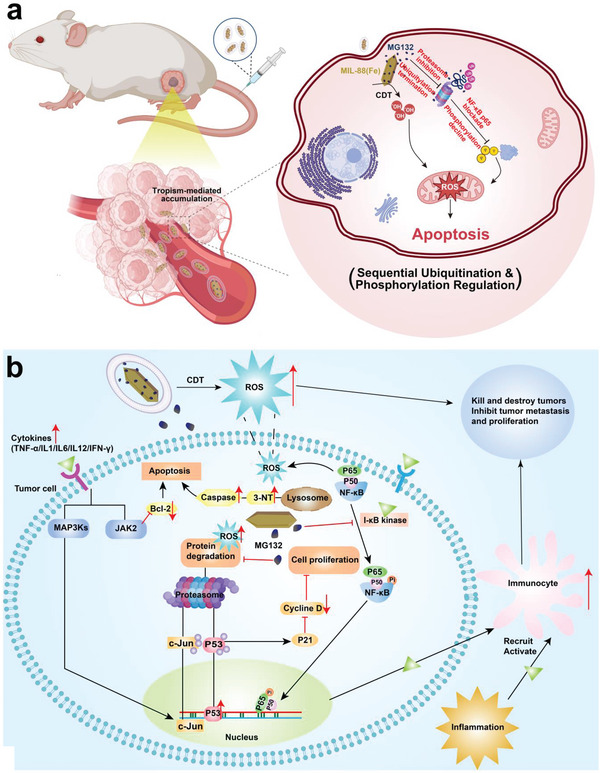
Principle and singling pathways of such sequential ubiquitination and phosphorylation epigenetics regulation strategy based on such MIL‐88‐MG132@M nanoplatforms. a) Schematic image of CDT and sequential ubiquitination and phosphorylation epigenetics regulation; b) The underlying singling pathways of such ubiquitination and phosphorylation‐engineered CDT‐based nanoplatforms for magnifying oxidative stress and repulsing mCRC.

It has been documented that 26S proteasome as the multi‐catalytic enzyme complexes can mediate proteolytic degradations of many pro‐apoptotic proteins such as p53, NF‐*κ*B, and cyclins.^[^ ^36^, ^37^, ^38^ ^]^ Intriguingly, many commercially‐available proteasome inhibitors that present pioneering and impressive anti‐tumor outcomes encourage us to construct this epigenetics regulation‐based CDT nanoplatform.^[^ ^39^, ^40^ ^]^ Inspiringly, the encapsulated 26S proteasome inhibitor in MIL‐88(Fe) CDT nanoplatform, that is, MG132 featuring high efficiency and cell permeability,^[^ ^41^, ^42^ ^]^ is anticipated to blockade the UPS‐mediated degradation of misfolded toxic proteins and disrupt tumor homeostasis since proteasome inhibitor alone can induce apoptosis via enriching ROS (**Figure** **1**a).^[^ ^43^, ^44^, ^45^ ^]^ In light of the fact that proteasome blockading for promoting apoptosis matters ROS accumulation,^[^ ^46^, ^47^ ^]^ the MG132‐mediated ubiquitination blockade is also expected to further blockade the phosphorylation of NF‐*κ*B transcription factors in mCRC, silence the carcinogenic driving genes associated with mCRC, which, thus, further magnifies the CDT‐incited oxidative stress (Figure 1a). Protein and RNA sequencing results verified the feasibility and role of such a sequence ubiquitination and phosphorylation epigenetics regulation in magnifying ROS and disarming resistance to oxidative stress. More significantly, NF‐*κ*B inactivation, UPS blockade, and MAPK and p53 activation are highlighted as the signaling pathways to manipulate this process (Figure 1b).

With collaborating with CDT, proteasome blockading‐mediated sequential ubiquitination and phosphorylation epigenetics modulation allowed the MG132‐loaded nanoplatforms (MIL‐88‐MG132) to harvest a considerably‐elevated antitumor outcome against highly‐aggressive mCRC. Notably, the excellent anti‐tumor outcome is probably in part attributed to the activated immune responses since epigenetics modulation also benefits immunotherapy.^[^ ^48^, ^49^ ^]^ Especially, macrophage membrane featuring inflammation tropism was coated on MIL‐88‐MG132@M, allowing more epigenetics‐engineered nanoplatforms to actively enter and retain in the tumor due to the presence of inflammation microenvironment, which thereby elevated the sequential epigenetics modulation enhanced CDT outcome again.^[^ ^50^ ^]^ Collectively, this sequential ubiquitination and phosphorylation epigenetics modulation strategy addressed the complex TME‐arised resistance to ROS fundamentally and opened up another avenue to enhance the anti‐tumor treatment efficiency of all ROS‐based treatment methods, such as SDT and PDT, which will arouse increasing interests among different communities.

## 2 Results and Discussion

### 2.1 Synthesis of MIL‐88‐MG132 and MIL‐88‐MG132@M

**Figure 2:**
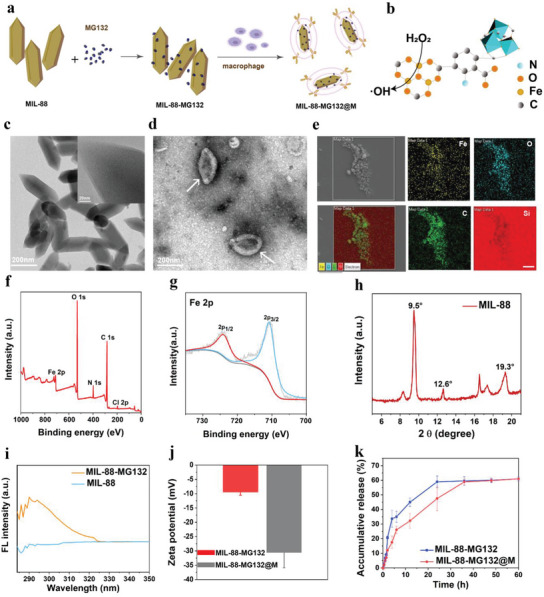
Synthesis and characterizations of MIL‐88‐MG132@M nanoparticles. a) The synthesis routine of MIL‐88‐MG132@M nanoparticles. b) Schematic of MIL‐88 structure and the mediated CDT process. c) Transmission electron microscopy (TEM) image of MIL‐88 nanoparticles. d) TEM micrograph of MIL‐88 nanoparticles after negative staining. e) Element mapping of Fe, O, C, and Si of MIL‐88 nanoparticles. f,g) Wide‐window X‐ray photoelectron spectroscopy (XPS) spectrum (f) and Fe2p XPS spectrum (g) of MIL‐88, indicating the valence of Fe, +3. h) X‐Ray powder diffraction pattern of the MIL‐88 nanocrystal. i) UV–vis spectra of MIL‐88 and MIL‐99‐MG132. j) Zeta potentials of MIL‐88‐MG132 and MIL‐88‐MG132@M. k) Release profiles of MG132 from MIL‐88‐MG132 or MIL‐88‐MG132@M. Data are expressed as mean ± standard deviation (SD) (*n* = 3).

Amino groups‐modified MIL‐88 with Fe as center atoms were first synthesized, followed by sequential MG132 entrapment and macrophage membrane coating (**Figure** **2**a), respectively, through which MIL‐88‐MG132 and MIL‐88‐MG132@M are easily accessible. The structure and CDT process of MIL‐88(Fe) is indicated in Figure 2b, wherein the organic covalent skeleton is clearly found. Herein, regular spindle‐structured nanoparticles with averaged 200 nm in diameter are obtained with high dispersity (Figure 2c,d and Figure S1, Supporting Information).

Energy diffraction spectrum and atom mapping indicate the presence of Fe action centers (Figure 2e and Figure S2, Supporting Information), suggesting the successful synthesis of MIL‐88(Fe) with invariable valence (+3, Figure 2f,g) and high crystallinity (Figure 2h), which also denotes the stable structure and high catalytic activity. Depending on the porous structure (Figure S3, Supporting Information), MG132 entrapment by NH_2_‐MIL‐88(Fe) MOF is within easy reach to obtain MIL‐88‐MG132. The emergence of a clear UV–vis characteristic peak of MG132 in MIL‐88‐MG132 adequately indicates the successful MG132 entrapment in comparison to MIL‐88(Fe) alone (Figure 2i). A similar result is obtained in the monitoring of FTIR spectra, where a new vibration peak at around 2830 cm^−1^ is obtained in MIL‐88‐MG132 (Figure S4, Supporting Information). To determine the optimal feeding ratio, different loading capabilities of MG132 in MIL‐88‐MG132 were traced, and a 1:1 feeding ratio of MIL‐88/MG132 is desirable under which the highest loading capacity (≈48%) is obtained (Figure S5, Supporting Information). Due to the modification of amino groups, MIL‐88(Fe) MOF is positively charged (Figure 2j), which is favorable for membrane coating via electrostatic interactions since the macrophage membrane is usually negatively charged. In detail, the zeta potential is reversed from positively‐charged MIL‐88‐MG132 to negatively‐charged MIL‐88‐MG132@M (Figure 2j). Accordingly, the particle size increases from MIL‐88‐MG132 to MIL‐88‐MG132@M (Figure S6, Supporting Information). The variations of Zeta potential and particle size provide powerful evidence to verify the macrophage membrane coating.

Gel results unveil that no protein degradation or structure variation on the macrophage membrane is observed during the extraction and coating of the macrophage membrane, indicating the intact retention of macrophage membrane proteins (Figure S7, Supporting Information). This phenomenon means the function preservations of membrane protein, dictating the feasibility of targeting the delivery of MIL‐88‐MG132@M into tumors via the intratumoral inflammation tropism or homing mediated by macrophage membrane proteins. Despite delaying MG132 release at the early stage, the macrophage membrane coating fails to eventually affect the accumulative rate of MG132 from MIL‐88‐MG132@M within 60 h (Figure 2k). The release mechanism is a common concentration gradient‐driven free diffusion rather than acidic TME‐triggered release since low pH fails to significantly vary the release profile of MG132 from MIL‐88‐MG132@M (Figure S8, Supporting Information). Moreover, the high structural and colloidal stabilities in different media with variable pH values ensure long‐term storage and applicability (Figure S9, Supporting Information). Such MIL‐88‐MG132@M nanoplatforms are expected to improve the bioavailability of insoluble MG132, protect MG132 against degradation or decomposition, avoid leakage‐arised side effects, prolong the half‐life, and allow more nanoplatform to accumulate in the tumor. All these appealing outcomes will benefit the sequential ubiquitination and phosphorylation epigenetics regulation and hamper the expressions of carcinogenic driving genes in mCRC, which is favorable for magnifying CDT outcomes against mCRC.

### 2.2 CDT Process Uniting with Sequential Ubiquitination and Phosphorylation Epigenetics Reshaping for ROS Production

**Figure 3:**
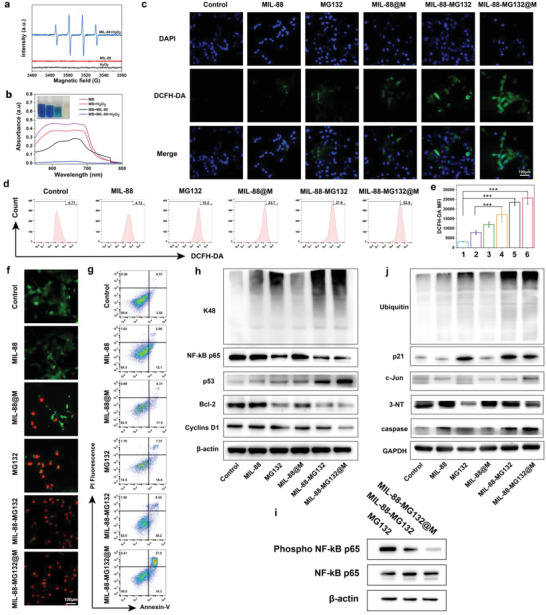
In vitro evaluations on CDT‐mediated ROS production, antitumor properties, and the sequential ubiquitination and phosphorylation epigenetics regulation principle based on the MIL‐88‐MG132@M nanoplatforms. a) ESR spectra of different groups (i.e., MIL‐88+H_2_O_2_, MIL‐88, and H_2_O_2_) for detecting ∙ OH. b) UV–vis spectra of MB aqueous solution after degradation by different treatments for 1 h. c) CLSM images of CT‐26 cells after different corresponding treatments and subsequent DCFH‐DA staining. d,e) FCM patterns (d) and the corresponding fluorescence intensities (e) of ROS‐indicator‐stained CT‐26 cells for tracking ROS levels after different treatments. f,g) CLSM images (f) and FCM patterns (g) of CT‐26 cells co‐stained by calcein AM/PI after different corresponding treatments. h–j) In vitro WB bands of various proteins collected from CT‐26 cells after different treatments. Data are expressed as mean ± SD (*n* = 3). One‐way analysis of variance (ANOVA) was used to test the difference significance, and ***p < 0.001.

In the course of CDT, multivalent metal ion‐mediated Fenton or Fenton‐like reactions were carried out to catalytically decompose H_2_O_2_ into highly‐toxic hydroxyl radicals (∙OH), resulting in strong oxidative damage to cancer cells in situ.^[^ ^16^ ^]^ In this regard, Fe ion centers in MIL‐88 are expected to in‐situ generate ∙OH in the TME. The typical electron spin resonance (ESR) peaks of ∙OH after H_2_O_2_ incubation with MIL‐88 (i.e., MIL‐88+H_2_O_2_) are observed, while no ESR peaks are found in either H_2_O_2_ alone or MIL‐88 alone, suggesting the occurrence of MIL‐88‐mediated chemodynamic process in MIL‐88+H_2_O_2_ (**Figure** **3**a). The absorbance intensity of methylene blue (MB) peaking at 665 nm was monitored to further assess the chemodynamic process‐derived ROS level. Intriguingly, similar results are obtained, where the MIL‐88+H_2_O_2_ group receives the largest drop magnitude of signal intensity (Figure 3b), suggesting that the MIL‐88‐mediated Fenton reaction process successfully decomposed H_2_O_2_ and produced the most ∙OH to oxidize and degrade MB. As the incubation time is prolonged, more ∙OH can be produced (Figure S10, Supporting Information). Remarkably, the blue MB solution is transformed into a colorless one (insets in Figure 3b), further demonstrating MB degradation by MIL‐88‐mediated CDT‐arised hydroxyl radicals. All of the above showed that MIL‐88 has strong catalytic properties, which laid an important foundation for the subsequent CDT against tumors.

Besides the direct ROS production by CDT, reducing ROS depletion or inducing ROS accumulation after combining with other approaches was highly encouraged.^[^ ^32^, ^51^ ^]^ In the ubiquitin‐proteasome degradation pathway, intracellular target protein tagging with ubiquitin chains and proteasome‐enabled endocytosis and hydrolysis are two dominant rate‐limiting steps, both of which manipulate the proliferation, differentiation, cycle, and apoptosis of cells.^[^ ^52^, ^53^ ^]^ Regarding this, blockading proteasome‐mediated hydrolysis can be regarded as one pathway for heightening ROS‐based anti‐tumor efficiency. Herein, the proteasome inhibitors (MG132) were introduced to induce apoptosis and promote ROS accumulation via blockading UPS‐mediated degradation of pro‐apoptotic proteins. Furthermore, MG132 can inhibit the phosphorylation of transcription factors and silence the carcinogenic driving genes in mCRC, which will further contribute to ROS accumulation. To understand it, the intracellular ROS level was traced by confocal laser scanning microscopy and flow cytometry (FCM) (Figure 3c–e), respectively, where mCRC CT‐26 cells treated with different groups were stained by the ROS indicators (i.e., 2,7‐dichlorodihydrofluorescein diacetate [DCFH‐DA]).^[^ ^51^ ^]^ It is clearly found that MIL‐88 alone and MG132 alone promote ROS to rise via Fenton reaction‐mediated CDT process and the ubiquitination and phosphorylation epigenetics regulations, respectively. Once the two components are combined, the CDT process‐arised ROS production and the dual‐epigenetics regulation‐induced ROS accumulation in MIL‐88‐MG132 synergize to elevate the ROS level. Inspiringly, the macrophage membrane coating further allows more NPs to enter CT‐26 tumor cells through active tropism (Figure S11, Supporting Information), which enables MIL‐88‐MG132@M to exert more robust actions to acquire higher ROS content. The higher MIL‐88‐MG132@M NPs concentration is added, and more ROS production is attained (Figure S12, Supporting Information).

### 2.3 CDT Process Uniting with Sequential Ubiquitination and Phosphorylation Epigenetics Reshaping for Promoting Cell Deaths

Inspired by the above massive ROS accumulation, the CDT process and sequential ubiquitination and phosphorylation epigenetics reshaping bring about the most cell deaths (Figure S13, Supporting Information). To further investigate their killing actions against tumor cells, dead/live cells were discerned by CLSM after propidium iodide (PI) and calcein AM co‐staining post‐different treatments. Results show that most cell deaths occur in the combined group with CDT, sequential ubiquitination and phosphorylation epigenetics reshaping, and macrophage membrane targeting (i.e., MIL‐88‐MG132@M), as indicated in Figure 3f. In this group, macrophage membrane tropism facilitates proteasome inhibitors (MG132) to be delivered into CT‐26 cells and recognized by aberrant components, inhibits ubiquitylation‐tagged toxic protein degradations and blockades NF‐*κ*B transcription factor phosphorylation, which eventually successfully amplifies the tumor‐killing effect of CDT‐rooted ROS in situ. Furthermore, FCM analysis proceeded after calreticulin acetylmethoxy/propidium iodide (calreticulin‐AM/PI) staining. Identical results are obtained, where the MIL‐88‐MG132@M group expedites the apoptosis rate of CT‐26 cells to significantly ascend (Figure 3g). Besides CT‐26 cell line, such CDT process and sequential ubiquitination and phosphorylation epigenetics reshaping also serve as a general method to induce ROS accumulation and cell deaths in other tumor cell lines, for example, HCT‐116 human colorectal cell (Figures S14 and S15, Supporting Information).

Apart from inducing cell deaths, the proliferation and migration activities of tumor cells were tracked. Herein, we moved forward to implement the cell scratch test on how the nanotherapeutic platform affects the migration force of CT‐26 tumor cells. After 48 h observation, the proliferation and migration of both tumor cell lines are significantly inhibited by the combined effect of MIL‐88 and MG132 relative to the control group (Figure S16, Supporting Information). This result uncovers that the combined effect of CDT and sequential ubiquitination and phosphorylation epigenetics reshaping merits to be highlighted to prevent tumor migration.

In order to further demonstrate the generality of such MIL‐88‐MG132@M nanotherapeutic platforms, a non‐colorectal tumor cell line, that is, liver tumor Hepa 1–6 was used, and similar results akin to CT‐26 cells or HCT‐116 cells were obtained. In detail, the CDT combined with the epigenetic modulation treatment group (i.e., MIL‐88‐MG132@M) is found to produce the highest ROS (Figure S17, Supporting Information) and induce the most cell deaths (Figures S18 and S19, Supporting Information), further validating the general applicability of this sequential ubiquitination phosphorylation combined with CDT treatment strategy in repressing tumor.

### 2.4 Sequential Ubiquitination and Phosphorylation Epigenetics Reshaping Evaluation

To figure out the principle of sequential ubiquitination and phosphorylation epigenetics reshaping for promoting tumor cell apoptosis based on MIL‐88‐MG132@M, we studied the molecular mechanisms by western blotting (WB). Obviously, the proteasome function is inhibited by MG132‐contained groups, as evidenced by the up‐regulation of related representative proteins (e.g., Ubiquitin and K48) and substrate proteins (e.g., p21 and c‐Jun) in the UPS pathway (Figure 3h,j and Figure S20, Supporting Information), wherein the highest expressions emerge in the MIL‐88‐MG132@M group. Herein, proteasome‐mediated protein degradation inhibition adequately disrupted the homeostasis, and enriched toxic misfolded proteins to activate c‐Jun N‐terminal kinase (Figure 3j and Figure S20, Supporting Information), ultimately leading to tumor cell apoptosis. This result validates the successful ubiquitination blockading that disables the degradation of toxic proteins. Anti‐apoptotic proteins (e.g., Bcl‐2, Cyclin D1) are significantly decreased (Figure 3h and Figure S20, Supporting Information), while cancer suppressor protein (e.g., p53) and oxidative stress‐associated protein (e.g., caspase family) are highly expressed (Figure 3h,j and Figure S20, Supporting Information), which contributed to the significantly‐elevated cell deaths mentioned above.

It has been documented that NF‐*κ*B activity in tumor cells is higher than that in normal cells, and it correlates with tumor proliferation‐activated genes and chronic inflammation progression‐related genes, which benefit tumor growth.^[^ ^54^ ^]^ In the classic pathway, the UPS system can liberate NF‐*κ*B from the NF‐*κ*B/IkBa complexes and promote IkBa phosphorylation. Regarding this, inhibiting proteasome function using such ubiquitination‐engineered effectors is also expected to stabilize NF‐*κ*B/IkBa complexes in the cytoplasm, suppress NF‐*κ*B activity and blockade its phosphorylation, which will prevent the development of chronic inflammation in TME and disfavor tumor progression. As expected, the expression level of NK‐*κ*B p65 protein is significantly down‐regulated by those MG132‐contained groups (Figure 3h and Figure S20, Supporting Information), and the multiple actions originating from MIL‐88‐MG132@M cause the lowest NK‐*κ*B p65 expression.

To exclude the interference of NK‐*κ*B p65 down‐regulation on NK‐*κ*B p65 phosphorylation assessment, the doses of MIL‐88‐MG132 and MIL‐88‐MG132@M were adjusted so as to make the expression of NK‐*κ*B p65 coincide with that using MG132 alone (Figure 3i). The phosphorylation of p65 Ser536 has been accepted to promote the nuclear translocation of p65, bind to caspase and Bcl‐2 promoters, enhance angiogenesis, and inhibit apoptosis.^[^ ^54^ ^]^ An inspiring phenomenon is observed that the phosphorylation of NF‐*κ*B‐p65 is remarkably inhibited in the MIL‐88‐MG132@M group wherein the rich ROS production exerts the preferable inhibitory action (Figure 3i). Taken all above together, the successfully‐established sequential ubiquitination and phosphorylation epigenetics regulation strategy not only contributed to cutting off nutrients and oxygen supply to the proliferative tumor cells but also acted as a repressor for hampering migration.

### 2.5 In Vivo Antitumor Assessment Based on CDT and Sequential Ubiquitination and Phosphorylation Epigenetics Reshaping

**Figure 4:**
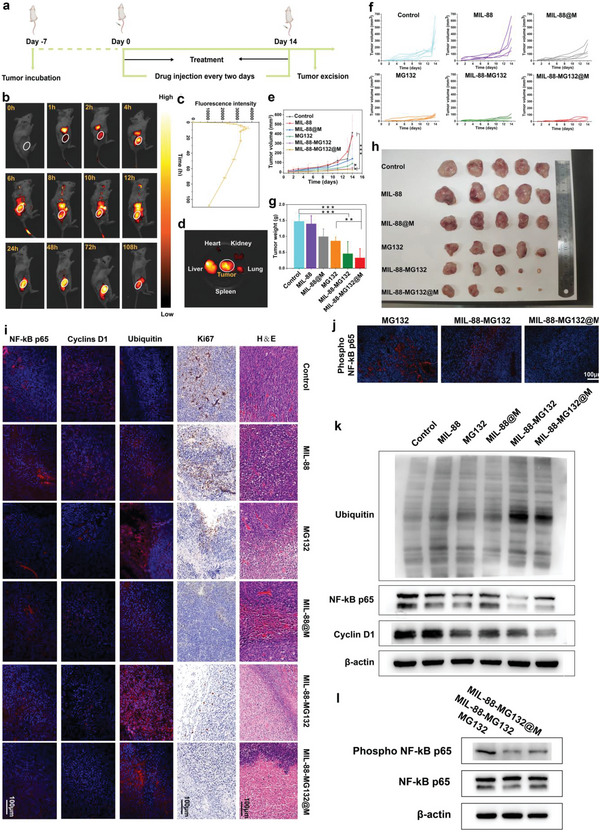
In vivo anti‐tumor evaluations and the explorations on the sequential ubiquitination and phosphorylation epigenetics regulation using the MIL‐88‐MG132@M nanoplatforms. a) Time axis of in vivo anti‐tumor experiments and operation details. b,c) Time‐dependent fluorescence images (b) and signal intensities (c) of CT‐26 tumor‐bearing mice after intravenous administration of IR780‐labeled MIL‐88‐MG132@M; and d) ex vivo fluorescence images of the tumor tissues and other major organs harvested from CT‐26 tumor‐bearing mice after 6 h post‐intravenous administration of IR780‐labeled MIL‐88‐MG132@M. e) Time‐dependent averaged tumor volume of CT‐26 in CT‐26 tumor‐bearing mice that experienced different treatments in different groups; f) Detailed tumor‐growth curves of each mouse per group after different treatments including control, MIL‐8, MG132, MIL‐8@M, MIL‐88‐MG132, MIL‐88‐MG132@M. g,h) Weights (g) and digital photographs (h) of dissected tumors isolated from HCT116 tumor‐bearing mice that experienced corresponding treatments in different groups. i) Immunohistochemical and immunofluorescence images of tumor slices isolated from CT‐26 tumor‐bearing mice that experienced corresponding treatments in different treatment groups, including HE, Ki67, NF‐*κ*B p65, Cyclins D1, and Ubiquitin. j) Phospho NF‐*κ*B p65 immunofluorescence images of tumor slices isolated from CT‐26 tumor‐bearing mice that experienced corresponding treatments in different treatment groups. k,l) WB bands of various proteins collected from CT‐26 tumor implanted on tumor‐bearing mice that experienced corresponding treatments in different treatment groups. Data are expressed as mean ± SD (*n* = 5). Student's *t*‐test was used to test the difference significance between the two groups, and ***p < 0.001.

Inspired by the above appealing and exciting results, in vivo anti‐tumor efficacy using the combined actions consisting of CDT, sequential ubiquitination and phosphorylation epigenetics reshaping, and macrophage membrane targeting were evaluated, and the experimental procedures are shown in **Figure** **4**a. In vivo bio‐distribution tests show that macrophage membrane coating prolongs the circulation of MIL‐88‐MG132@M NPs, improves their retention level, and sustains a long residence time in mCRC CT‐26 tumor, as indicated by the comparison of time‐dependent fluorescence images and intensities at the site of tumor between MIL‐88‐MG132@M (Figure 4b,c) and MIL‐88‐MG132 (Figure S21, Supporting Information) groups. The ex vivo fluorescence image of the isolated tumor and other main organs also indicates the massive accumulation of MIL‐88‐MG132@M in mCRC CT‐26 tumor (Figure 4d), and the high and prolonged retention of MIL‐88‐MG132@M in tumor preferably ensures the in vivo anti‐tumor outcomes. After monitoring the time‐correlated tumor volumes, the treatment with MIL‐88 alone fails to inhibit tumor growth probably due to the poor and short retention of MIL‐88, while macrophage membrane coating allows more MIL‐88@M to accumulate in tumor and induce robust CDT against mCRC tumor (Figure 4e,f). Especially, MG132 alone also can delay tumor progression, which receives the largest inhibitory effect against mCRC tumors after further combining with MIL‐88‐arised CDT or/and macrophage membrane‐mediated tropism accumulation (Figure 4e,f). At the end of the experimental period, the isolated CT‐26 tumors were weighed, and the tumor weight is tremendously shrunk in MIL‐88‐MG132 and MIL‐88‐C@M groups (Figure 4g,h), which further confirms that the MG132‐arised sequential ubiquitination and phosphorylation epigenetics reshaping can enhance MIL‐88‐mediated CDT of the malignant tumor. During the treatment, no body fluctuation is observed (Figure S22, Supporting Information), and it also poses no damage to normal organs (Figure S23, Supporting Information).

Pathological examinations proceeded. Ki67 and H&E results indicate that inhibited cell proliferation and promoted cell deaths are responsible for the most remarkable tumor shrinkage (Figure 4i). Further immunofluorescence observations uncover the down‐regulated expressions of Cyclin D1 and NF‐*κ*B p65 and the up‐regulated Ubiquitin when the CT‐26 tumor‐bearing mice underwent MG132‐contained treatments (Figure 4i), akin to in vitro results. In particular, phosphorylated NF‐*κ*B p65 is also found to be down‐regulated (Figure 4j). Similar results are obtained via WB analysis, where more Ubiquitin expression is found and the expression levels of Cyclin D1, NF‐*κ*B p65, and Phospho NF‐*κ*B p65 evidently descend (Figure 4k,l and Figure S24, Supporting Information). All these results adequately demonstrate the sequential ubiquitination and phosphorylation epigenetics reshaping at the in vivo level and explain why such ubiquitination and phosphorylation‐engineered effectors harvested the incredible CDT outcome against mCRC tumor. More significantly, biochemical indexes and liver and kidney indexes show no evident fluctuations (Figures S25 and S26, Supporting Information), and even higher concentrations also fail to do damages normal organs and body weight of mice (Figure S27 and S28, Supporting Information), suggesting the excellent biosafety of MIL‐88‐MG132@M.

### 2.6 Activated Immune Response Assay

**Figure 5:**
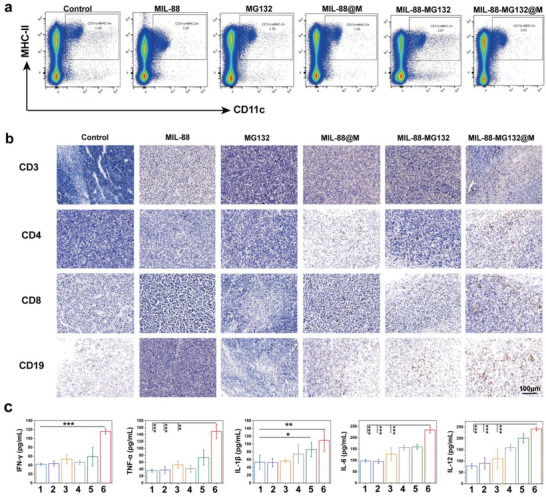
Detection of tumor‐associated immunity. a) Representative FCM data of matured DCs in the spleen of CT‐26 tumor‐bearing mice after different corresponding treatments in groups 1–6, and matured DCs were gated from the live CD11c+ /MHC‐II+ cells in the spleen. b) Immunohistochemical examination of CD3, CD4, CD8, and CD19 in CT‐26 tumor after different treatments in groups 1–6. c) Expression levels of pro‐inflammatory cytokines in primary tumors after different corresponding treatments in groups 1–6. Note, groups 1–6 represent Control, MIL‐88, MG132, MIL‐88‐MG132, and MIL‐88‐MG132@M, respectively. Data are expressed as mean ± SD (*n* = 5). Student's *t*‐test was used to test the difference significance between the two groups, and *p <0.05, **p < 0.01, and ***p <0.001.

Besides direct CDT and sequential ubiquitination and phosphorylation regulation‐enhanced CDT, activated immune responses are in part responsible for the mCRC recession in reality. To determine it, we assessed the changes in immune cells and cytokines in TME. Dendritic cells (DCs) isolated from the spleens were traced using FCM. Compared with other control groups, the highest expression levels of CD11c+MHC‐II+ are found in the MIL‐88‐MG132@M group (**Figure** **5**a and Figure S29, Supporting Information), which may be related to the fact that MIL‐88‐MG132@M treatment triggered abundant tumor‐associated antigens to stimulate DCs and present them to lymphocytes, enabling the proliferation and activation of antigen‐specific T and B lymphocytes to initiate adaptive immune responses. Accordingly, the infiltrated cytotoxic T‐lymphocytes (CTLs, CD3+CD8+) and CD3+CD4+ T cells are increased (Figure 5b), and MIL‐88‐MG132@M treatment receives the highest CTLs recruitment and the highest IFN‐*γ* expression (Figure 5c), implying the presence of a strong CTL‐mediated anti‐tumor immune effect.^[^ ^55^ ^]^ In light of the fact that CD4+ T cells can enhance phagocyte‐mediated anti‐infective effects and B cell‐mediated humoral immune response,^[^ ^55^ ^]^ CD19+ expression also exhibits an escalation trend (Figure 5b). Notably, MIL‐88‐MG132@M causes the highest CD19+ content and the highest secretion levels of IL‐1*β*, TNF‐*α*, IL‐12, and IL‐6 (Figure 5c), which may be associated with enhanced B cell‐mediated humoral immune response. Based on these results, we consider that the treatment in MIL‐88‐MG132@M switched tumor‐supportive chronic inflammation to immune activation‐related acute inflammation in TME, which thereby inhibited tumor cell proliferation.

### 2.7 RNA Sequencing for Determining Signaling Pathways

**Figure 6:**
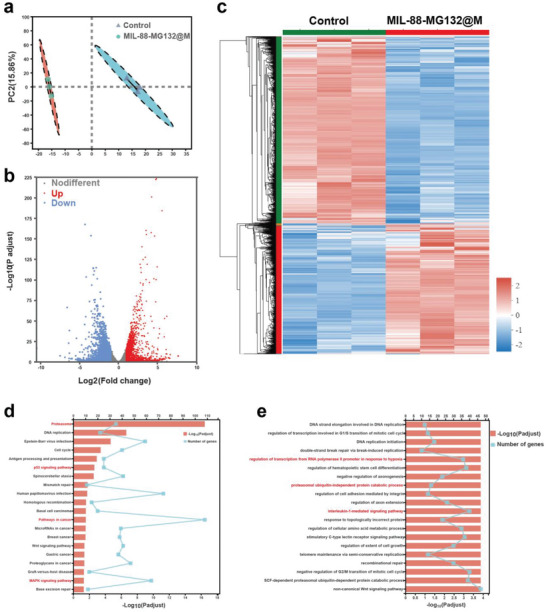
RNA sequencing for analyzing CDT combined with inhibition of ubiquitin‐proteasome pathway in MIL‐88‐MG132@M. a) PCA analysis of CT‐26 cells in the control group and MIL‐88‐MG132@M group. b,c) Volcano map (b) and heat map (c) of total differential genes in CT‐26 tumor between control and MIL‐88‐MG132@M groups (*p* < 0.05, |fold change| ≥ 2). d) KEGG analysis of differential gene expression profiles based on RNAseq after the MIL‐88‐MG132@M treatment, which is available for uncovering the affected pathways by the above differential genes. e) GO analysis of differential gene expression profiles based on RNAseq after the MIL‐88‐MG132@M treatment.

To deeply understand how the sequential ubiquitination and phosphorylation epigenetics reshaping disarms resistances to ROS and magnify ROS anti‐tumor effect, RNA sequencing was carried out. According to the macroscopic examination including the principle component analysis (PCA) and expression difference statistics (volcano plot and heat map), the treatment group has a prominent difference in gene expressions, as shown in **Figure** **6**a **–c**. In comparison to the control group (Figure 6b,c), MIL‐88‐MG132@M induces 1527 up‐regulated genes and 2198 down‐regulated genes (*p* < 0.05, log2FoldChange|>1), respectively. In detail, the Kyoto Encyclopedia of Genes and Genomes (KEGG) analysis indicates that MIL‐88‐MG132@M treatment correlates with the UPS pathway (Figure 6d), wherein p53 and MAPK pathways are activated. GO enrichment analysis reveals that the inspiring anti‐tumor outcome of MIL‐88‐MG132@M treatment regulates several pathways (Figure 6e), for example, the transcriptional control of RNA polymerase II promoter toward anoxia, proteasome‐involved metabolism, NK‐*κ*B inactivation, and IL‐1‐mediated immunity. These results validate that MIL‐88‐MG132@M treatment exerted excellent anti‐tumor activity mainly through UPS pathway interference, oxidative stress provocation, and NK‐*κ*B blockade.

Taken above WB and RNA sequencing data, the potential pathway using such a sequential ubiquitination and phosphorylation regulation effector to enhance CDT‐mediated oxidative stress for repressing tumors is summarized in Figure 1b. In brief, MIL‐88(Fe) in MIL‐88‐MG132@M first triggered the CDT process to produce ROS, during which MG132 was released to blockade the UPS pathway, inhibit ubiquitination, and enrich toxic misfolded proteins, followed by NF‐*κ*B pathway inactivation and MAPK and p53 pathways activation, establishing the sequential ubiquitination and phosphorylation epigenetics regulation. The two sequential epigenetics regulations broke tumor homeostasis and boosted oxidative stress, which eventually magnified the CDT‐arised ROS retention and induced the necrosis and apoptosis of tumor cells.

## 3 Conclusions

In summary, we developed a sequential ubiquitination and phosphorylation epigenetics regulation strategy based on the MIL‐88‐MG132@M nanoplatform to disarm the TME‐arised resistances to MIL‐88‐mediated CDT. The loaded MG132 was demonstrated to blockade proteasome and inhibit NF‐*κ*B p65 and its phosphorylation, which magnified oxidative stress. Multi‐omics analysis including proteomics and genomics uncovered the detailed signaling pathways, for example, UPS‐mediated toxic protein degradation blockade, NF‐*κ*B pathway inactivation, MAPK and p53 pathways activation, and acute inflammation‐arised immune response activation, all of which combined to contribute to the enhanced oxidative stress accumulation for repulsing mCRC. Additionally, macrophage membrane coating imparted the nanoplatforms with active tropism to intratumoral chronic inflammation, thereby improving the accumulation of MIL‐88 and MG132 to maximally propel the mCRC recession. This sequential dual‐epigenetics‐engineered CDT‐based nanoplatform holds high clinical translation and will provide more inspiration or insights into the development of other ROS‐surging nanoplatforms.

## Conflict of Interest

The authors declare no conflict of interest.

## Author Contributions

Z.B., J.Y., and Y.Z. contributed equally to this work. K.Z. conceived this project; and K.Z., Z.B., and Y.Z. designed the experimental contents and plans. Z.B., J.Y., Y.Z., T.L., C.F., X.L., Q.P., N.L., and D.W. performed the experiments. K.Z., Z.B., and Y.Z. analyzed the data. K.Z. wrote and revised the original manuscript. W.T., K.Z., and J.Y. supported the project. K.Z. supervised the project and all authors commented on this manuscript.

## Supporting information

Supporting Information

## Data Availability

The data that support the findings of this study are available from the corresponding author upon reasonable request.
