# Supplementary material for: Sequential Ubiquitination and Phosphorylation Epigenetics Reshaping by MG132‐Loaded Fe‐MOF Disarms Treatment Resistance to Repulse Metastatic Colorectal Cancer
Source: Adv Sci (Weinh). 2023 Jun 11;10(23):2301638. doi: 10.1002/advs.202301638 (PMC10427397; doi:10.1002/advs.202301638)
Supplement: Supplementary file 1 — Supporting Information [file ADVS-10-2301638-s001.pdf]

ADVANCED SCIENCE
Open Access

# Supporting Information

for *Adv. Sci.*, DOI 10.1002/advs.202301638

Sequential Ubiquitination and Phosphorylation Epigenetics Reshaping by MG132-Loaded Fe-MOF Disarms Treatment Resistance to Repulse Metastatic Colorectal Cancer

*Zhaoting Bu, Jianjun Yang, Yan Zhang, Tao Luo, Chao Fang, Xiayi Liang, Qiuxia Peng, Duo Wang, Ningjing Lin, Kun Zhang* and Weizhong Tang**

**Sequential Ubiquitination & Phosphorylation Epigenetics Reshaping by MG132-Loaded Fe-MOF Disarms Treatment Resistance to Repulse Metastatic Colorectal Cancer**

*Zhaoting Bu, Jianjun Yang, Yan Zhang, Tao Luo, Chao Fang, Xiayi Liang, Qiuxia Peng, Duo Wang, Ningjing Lin, Kun Zhang and Weizhong Tang*

## Material and methods

### *Material*

$FeCl_3 \cdot 6H_2O$ and Pluronic F127 was acquired from Sinopharm Chemical Reagents Co., Ltd. 2-Aminoterephthalic acid ($NH_2$-BDC), anhydrous ethanol (EtOH, AR) and acetic acid (CH3COOH, AR) were purchased from Sigma-Aldrich (China). The anti-Bcl-2 antibody (ab32124), anti-Caspase-3 antibody (ab32351), anti-Cyclin D1 antibody (ab16663), anti-Ubiquitin (linkage-specific K48) antibody (ab140601), anti-NF-κB p65 antibody (ab32536) and Anti-GAPDH antibody (ab181602) were purchased from Abcam. Anti-p21 antibody (2947), anti-p53 antibody (2527), anti-c-Jun antibody (9165), anti-Phospho-NF-κB p65 antibody (3033) and anti-Ubiquitin antibody (3936) were bought from Cell Signaling Technology. CCK8 assay kit, calcein-AM/PI staining kit, ROS indicator (DCFH-DA), membrane and cytosol protein extraction kit (P0033), protease and phosphatase inhibitor cocktail (P1045), anti- $\beta$-actin antibody (AF0003), HRP-labeled goat anti-rabbit IgG (A0208), HRP-labeled goat anti-mouse IgG (A0216) and BCA protein quantification kit were obtained from Beyotime (Shanghai). Fetal bovine serum (FBS), roswell Park Memorial Institute (RPMI) 1640, dulbecco's modified eagle medium (DMEM), trypsin-EDTA Solution and penicillin-streptomycin solution were bought from Gibco (USA). Anti-3-nt antibody (GTX102509) was obtained from Gene Tex.

### *Characterizations*

Transmission electron microscopy (TEM) images, energy dispersive X-ray spectroscopy (EDS) element mappings of MIL-88 were obtained on JEM-2100F (Japan Electronics Co. LTD). MIL-88-MG132@M nanoparticles was observed using negative staining with phosphotungstic acid (JEOL JEM-1400Plus). Scanning electron microscopy (SEM) and corresponding elemental mapping were performed on field emission Magellan 400 microscope (FEI Company). X-ray photoelectron spectroscopy (XPS) was used to detect the valence state of Fe in MIL-88 nanoparticles by ESCAlab250 (Thermal Scientific). The XRD pattern was recorded on a D8 Advance diffractometer (Bruker, Germany). Fourier transform infrared (FTIR) spectra were obtained using a Nicolet iS 50 spectrometers (Thermo Scientific). The ultraviolet-visible (UV-Vis) absorption spectra were collected on a UV-3101 PC Shimadzu spectrometer. The hydrodynamic diameters and zeta potentials of MIL-88-MG132 and MIL-88-MG132@M nanoparticles were examined on a Malvern Zeta particle sizer (Nano ZS90, Malvern).

### *Preparation of MIL-88@M*

0.36 g of $FeCl_3 \cdot 6H_2O$ and 0.32 g of F127 were added to 26.68 mL of deionized water and the solution was vigorously mixed at room temperature for 2 h. Afterwards, 0.6 mL of acetic acid was added. After mixing for

another 1 h, 120 mg of 2-aminoterephthalic acid was injected. The mixture product was shaken for an additional 1 h. Then, the mixture was transferred to a 50 mL Teflon-lined autoclave and heated at 383 K for 24 h. After the reaction was completed, the obtained product was centrifuged at 8000 rpm for 10 min and then purified with ethanol several times to remove the residual reactants and surfactants. Finally, the dark brown solid product was dried at 333 K under vacuum for 4 h to obtain MIL-88. As for MG132 loading, MG132 (10 mM) was stirred with MIL-88 nanoparticles (5 mg/mL, 1 mL) for 24 h, followed by centrifugation and water washing 3 times to obtain MIL-88-MG132.

Raw.267 cells were cultured at 37 °C in the air atmosphere containing 5% $CO_2$. Raw.267 cells were cultured in DMEM. Raw.267 cell membrane proteins were extracted using Cell membrane protein extraction kit (Beyotime Biotechnology). After that, the extracted Raw.267 cell membranes were mixed thoroughly with MIL-88-MG132, and then MIL-88-MG132@M was obtained by repeatedly pushing it through a liposome extruder at 200 nm for 10-20 times in a filter. The final obtained MIL-88-MG132@M were stored at 4 ºC.

***In vitro drug releasing behavior of MIL-88-MG132@M***

MIL-88-MG132 methanol suspension (3 mL, 1 mg/mL) and MIL-88-MG132@M methanol suspension (3 mL, 1 mg/mL) were placed in dialysis bags (MWCO =3000), respectively, and then the dialysis bags were sealed to dialyze against another 250 mL of methanol solution in beakers, respectively. The experiments were carried out at 37 °C under continuous stirring. At different time points (0 h, 0.5 h, 1 h, 1.5 h, 2 h, 4 h, 6 h, 12 h, 24 h, 36 h, 48 h, 60 h), 1 mL of media was taken out to measure the released concentration by UV-Visible spectra on UV-3101PC Shimadzu spectrometer, wherein the peak intensity at 290 nm was recorded. Concurrently, 1 mL of fresh methanol was added to the solution so as to keep the constant volume.

***In vitro stability experiments***

The MIL-88-MG132 suspension (2 mL, 1 mg/mL) was placed in different solvents including PBS (pH=7.4), PBS (pH=5.0), DMEM, FBS, respectively. The morphology of MIL-88-MG132 nanoparticles were observed using transmission electron microscopy (TEM, JEOL 2011) for different durations (0 day, 3 day, 7 day). Correspondingly, the hydrodynamic diameters of MIL-88-MG132 nanoparticles were examined on a Malvern Zeta particle sizer (Nano ZS90, Malvern).

***Identification of membrane proteins on MIL-88-MG132@M by SDS-PAGE gel electrophoresis***

The detailed procedures were given as follows: Pouring the separation gel along the edge of the glass and sealing it with ultrapure water liquid; Allowing it to be solidified and removing the top layer of water; Filling the

concentrated gel and inserting a spotting comb; Mixing the macrophage membrane protein sample with various material with 5x protein loading buffer in a 4:1 ratio; and heating them in a 100 °C water bath for 10 min to denature the protein. After cooling, protein marker as parallel control was added to the spot sample wells. Electrophoresis was lunched under 80 V for about 45 min to allow protein samples and marker to enter the separated gel area. Subsequently, the voltage was adjusted to 120 V, during which each band was monitored. Electrophoresis was stopped when the bands were completely separated and travelled to the lower end of the electrophoresis tank. The gel was removed and placed in Komas Brilliant Blue staining solution. The gels were then placed on a shaker for more than 1 h. Afterwards, the gels were washed with ultrapure water, and then decolorized with acetic acid decolorization solution. The solution was changed at 2 h – 3 h intervals and the operation was repeated until the background was clean. The gels were videotaped for observation and labeling.

***ROS test***

In vitro •OH was measured on electron spin resonance (ESR) spectroscopy (JEOL-FA 200, Japan). Intracellular reactive oxygen species (ROS) levels were measured using flow cytometry (BD LStometry) after treatment. Confocal images were acquired by confocal laser scanning microscopy (CLSM 710; Carl Zeiss, Germany). The MIL-88 (0.5 mg/mL), $H_2O_2$ (10 mM), and MB (10 μg/mL) were prepared in deionized water; and the total volume was 1 mL. Then the reaction system was shaken at 37 ºC. At different time points, the mixture was centrifuged and the remaining MB in the supernatant was measured by UV/vis spectrophotometer at 664 nm. Additionally, MB (10 μg/mL) and MIL-88 (0.5 mg/mL) were shaken for different time points (0 h, 0.5 h, 1 h, 2 h, 4 h), and the absorbance at 660 nm were also recorded by UV-vis spectrometer.

As for CLSM observation, CT-26 cells, HCT-116 cells, or Hepa 1-6 cells were seeded into 20 mm confocal dishes ($1 \times 10^5$ cells per dish) and incubated overnight to allow them to be adhered to the wall, followed by DCFH-DA staining according to the assay kit instruction and corresponding treatments (including control, MIL-88, MG132, MIL-88@M, MIL-88-MG132, MIL-88-MG132@M). After 30 min, CLSM observation was then performed (Zeiss LSM880, Carl Zeiss, Germany).

***Cell culture***

CT-26 cells, HCT-116 cells, or Hepa 1-6 cells were cultured in DMEM or RPMI-1640 at 37 °C in air containing 5% $CO_2$; and RAW 264.7 were cultured in DMEM at 37 °C in air containing 5% $CO_2$. The cell medium was supplemented with 10% heat-inactivated FBS, 100 U/mL penicillin and 100 mg/mL streptomycin. The medium was replaced once per 2 days, during which the cells were digested by trypsin ad re-suspended in fresh DMEM or

RPMI-1640.

***Cellular uptake of MIL-88-MG132 and MIL-88-MG132@M***

MIL-88-MG132 or MIL-88-MG132@M engulfment by cells was observed using CLSM. CT-26 cells were seeded into 20 mm confocal dishes ($1 \times 10^5$ cells per dish) and incubated overnight to allow them to be adhered to the wall. Cells were then co-incubated with FITC-labeled MIL-88-MG132@M for 2 h, 4 h and 8 h, respectively. After washing with PBS 3 times, CLSM observation was performed (Zeiss LSM880, Carl Zeiss, Germany).

***Cytotoxicity tests***

CCK-8 assay was harnessed to investigate the in vitro cytotoxicity of different samples. In detail, CT-26 cells or Hepa 1-6 cells were inoculated into 96-well plates to maintain their density at $1\times10^4$ cells/well. Subsequently, the cells were treated by different groups (i.e., control, MIL-88, MG132, MIL-88@M, MIL-88-MG132, MIL-88-MG132@M), and culture medium were added to the wells and co-incubated with the cells for 24 hours. The relative cell viability was also detected using CCK8 assay method. 100 μL of CCK-8 solution (Beyotime Biotechnology, 10%) was added to each well and incubated for 1 h. Cell viability was measured using a microplate reader with an absorption peak at 450 nm.

***Calcein-AM/PI staining assay***

CT-26 cells, HCT-116 cells, or Hepa 1-6 cells were inoculated into 6-well plates and cultured overnight. After receiving different treatments (including control, MIL-88, MG132, MIL-88@M, MIL-88-MG132, MIL-88-MG132@M), cells were stained with Calcein-AM/PI solution at 37 ºC for 30 minutes. CLSM observation was then performed. In addition, the treated cells were stained with Annexin V-PI solution and analyzed by FCM.

***Western blot analysis in vitro***

CT-26 cells were inoculated in six-well plates and cultured until the cells reached the appropriate density. After different treatments (control, MIL-88, MG132, MIL-88@M, MIL-88-MG132, MIL-88-MG132@M), they were washed twice with 4 °C PBS. Subsequently, lysis solution was added and cells were lysed on ice for 30 min. Lysed products were collected and the supernatant was separated by centrifugation using a high-speed centrifuge (12,000 rpm, 5 min), and the total protein concentration of the supernatant was measured by the BCA protein assay kit. Equal amounts of total protein (20 μg) were electrophoresed on 10% SDS-polyacrylamide gel and transferred to PVDF membrane. Then the proteins on PVDF membranes were blocked by Blocking Buffer. After that, the primary antibody was incubated on the membrane overnight ar 4 °C. Subsequently, the primary antibody was removed by washing three times with TBST solution, and then the PVDF membrane was incubated with

conjugated secondary antibody (Beyotime, China) for 2 hours at room temperature. Finally, the ECL chemiluminescence kit was added dropwise to the PVDF membrane, and then the target proteins were visualized by the chemiluminescence/fluorescence image system (Tannon5200).

***Animal ethic approval***

Animals used in all experiments were supplied by the experimental animal center of Shanghai Tenth People's Hospital, and all animal experimental procedures were in accordance with the guidelines of the Animal Protection and Ethics Committee of the Shanghai Tenth People's Hospital with approval number (SHDSYY-2022-3429-202020728), Tongji University School of Medicine. As the tumor volume reached 1500 $mm^3$, the experimental was terminated.

***In vivo biosafety tests***

16 healthy female Bab/c mice (approximately 18 g) were randomly divided into four groups (n=5). Then the tail vein was injected with PBS, 88-MG132@M with varied concentrations (5 mg/kg, 10 mg/kg and 20 mg/kg), respectively, and then fed for 21 days to assess in vivo toxicity. Growth weights were recorded for 21 days, and after 21 days the mice were dissected and the major organs (heart, liver, spleen, lungs and kidneys) were collected, fixed in 10% paraformaldehyde and stained with hematoxylin and eosin (H&E). Thirty CT26 tumor-bearing Balb/c nude mice were randomly divided into 6 groups (n=5) with different therapies including (I) control, (II) MIL-88, (III) MG132, (IV)MIL-88@M, (V) MIL-88-MG132, (VI)MIL-88-MG132@M. Blood samples were collected at the end of 15 days of treatment for serum biochemical tests including platelet (PLT), white blood cell (WBC), red blood cell (RBC) , hemoglobin (HGB) , mean corpuscular volume (MCV) , hematocrit (HCT) , lymphocyte (LYM) , mean corpuscular hemoglobin (MCH), and mean corpuscular hemoglobin concentration (MCHC), alanine aminotransferase (ALT), aspartate aminotransferase (AST), total bilirubin (TBIL), creatinine (Crea), and urea. Major organs (heart, liver, spleen, lung, kidney and tumor) were collected and sliced for HE staining.

***In vivo biodistribution and metabolism of MIL-88-MG132@M***

The fluorescent dye IR780 was loaded in MIL-88-MG132@M and injected into CT26 tumor-bearing mice via tail vein. Fluorescence images were then collected from CT26 tumor-bearing nude mice at different time points (0 h, 0.5 h, 1.0 h, 1.5 h, 2 h, 4 h, 6 h, 8 h, 10 h, 12 h, 24 h and 48 h). Mice were dissected at 6 h after MIL-88-MG132@M /IR780 injection so as to observe the distributions of MIL-88-MG132@M /IR 780 in major organs.

***Evaluations on in vivo anti-tumor efficiency***

CT-26 cells dispersion in saline (100 μL) was injected in the right hind leg of each female Balb/c mouse (approximately 20 g) with approximately $1\times10^7$cells per mouse to establish the subcutaneous tumor model. All tumor-bearing nude mice were randomly divided into six groups (n=5), including 1. control, 2. MIL-88, 3. MG132, 4. MIL-88@M, 5. MIL-88-MG132, 6. MIL-88-MG132@M, where MIL-88-MG132@M dose was 20 mg/kg. The body weights and tumor sizes (length and width) of each mouse in the 6 groups were measured every other day. Tumor volumes were determined according to the formula: length×width×height×π/6. At the end of the treatment period, mice were dissected and their major organs (heart, liver, spleen, lungs, and kidneys) as well as tumors were harvested and fixed in 10% formalin. The tumors were then sliced and immunohistochemically stained with H&E, Ki67, Ubiquitin, Cyclins D1, NFκB-p65. WB analysis was in accord with above in vitro analysis.

***In vitro detection of DCs maturation***

The spleens of female Balb/c mice (8w) were collected, grinded and made into cell suspensions to attain DC cells. DC cells were co-incubated with different treatments for 12 h in groups 1-6, including 1. control, 2. MIL-88, 3. MG132, 4. MIL-88@M, 5. MIL-88-MG132, 6. MIL-88-MG132@M, respectively. After various treatments and another 12 h incubation, live cells were stained with DAPI and DCs were stained with anti-CD11c-APC, anti-MHC-II-FITC, respectively, and then instantly analyzed by FCM.

***In vivo detection pro-inflammatory cytokines***

Herein, CT-26 cells ($1\text{x}10^6$) were subcutaneously injected into the right frank of each BALB/c mouse (8 weeks) to construct a unilateral tumor model. Randomly, mice were divided into six groups (n = 5) named as 1. control, 2. MIL-88, 3. MG132, 4. MIL-88@M, 5. MIL-88-MG132, 6. MIL-88-MG132@M, respectively. Six days later, the tumors reached ~60 $mm^3$ before the experiments. MIL-88, MG132, MIL-88@M, MIL-88-MG132 and MIL-88-MG132@M were pre-dispersed in saline solution (dose: 20 mg/kg) and injected intravenously. On the $14^{th}$ day, tumors were excised and digested for CD3, CD4, CD8 and CD19 immunofluorescence staining. Meanwhile, the serum samples were collected after different corresponding treatments, and the pro-inflammatory cytokines, including TNF-α (70-EK282/4-48), IL-1β (70-EK201B/3-48), IL-6 (70-EK206/3-96), IL-12 (70-EK2183/2-48), IFN-γ (70-EK280/3-96) were measured by MULTISCIENCES (LIANKE) BIOTECH, CO., LTD, China) Mouse Inflammation Kit.

***RNA-sequencing***

Total RNA was isolated from TRCs using TRIzol reagent (Invitrogen, CA, USA) and the quantity and purity were monitored using NanoDrop ND-1000 (NanoDrop, Wilmington, DE, USA) as well as Bioanalyzer 2100 (Agilent,

CA, USA). OligodT magnetic beads (25-61005, Thermo Fisher, CA, USA) that could enrich mRNAs were fragmented. cDNAs were synthesized from the fragmented RNA using a Reverse Transcriptase (Invitrogen SuperScript II Reverse Transcriptase, CA, USA), and then sequenced by Illumina Novaseq 6000 (LC Bio Technology Co., Ltd. Hangzhou, China). Gene Ontology (GO) and Kyoto Encyclopedia of Genes and Genomes (KEGG) analyses were performed to identify potential pathways.

***Statistical analysis***

The quantitative data analysis was manifested as mean ± standard deviation (SD) (n=3~5). Statistical significance between the groups was determined by t-test or one-way analysis of variance (ANOVA). Values with $P < 0.05$ were considered statistically significant (*$P < 0.05$, **$P < 0.01$, ***$P < 0.001$, ****$P < 0.0001$).

## Supplementary figures

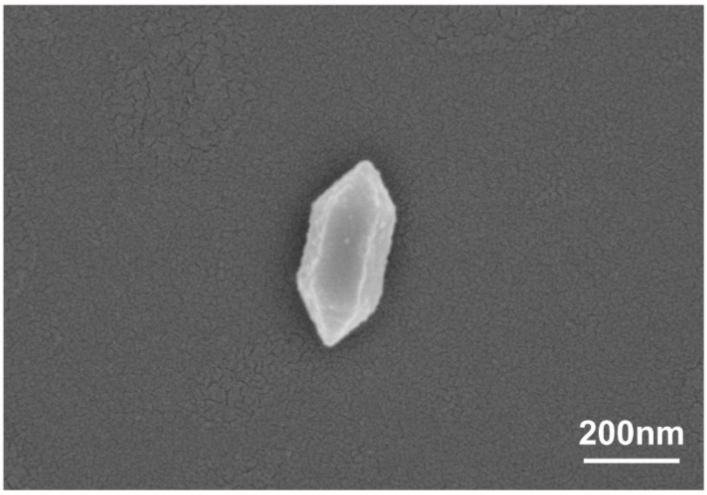


**Figure S1** SEM images of MIL-88.

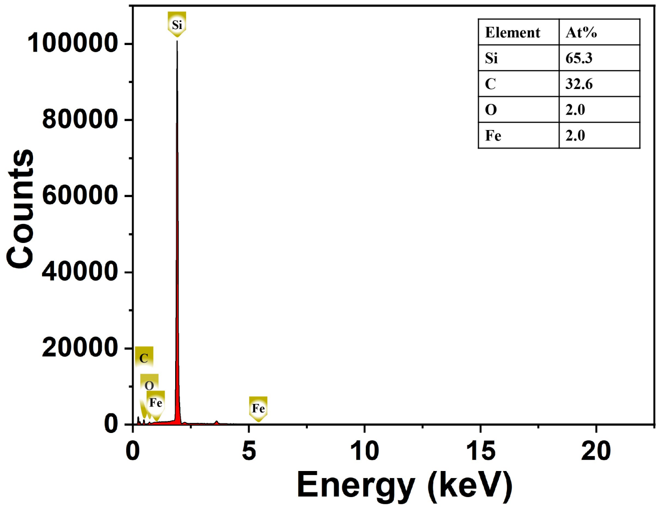


**Figure S2** EDS spectra of MIL-88.

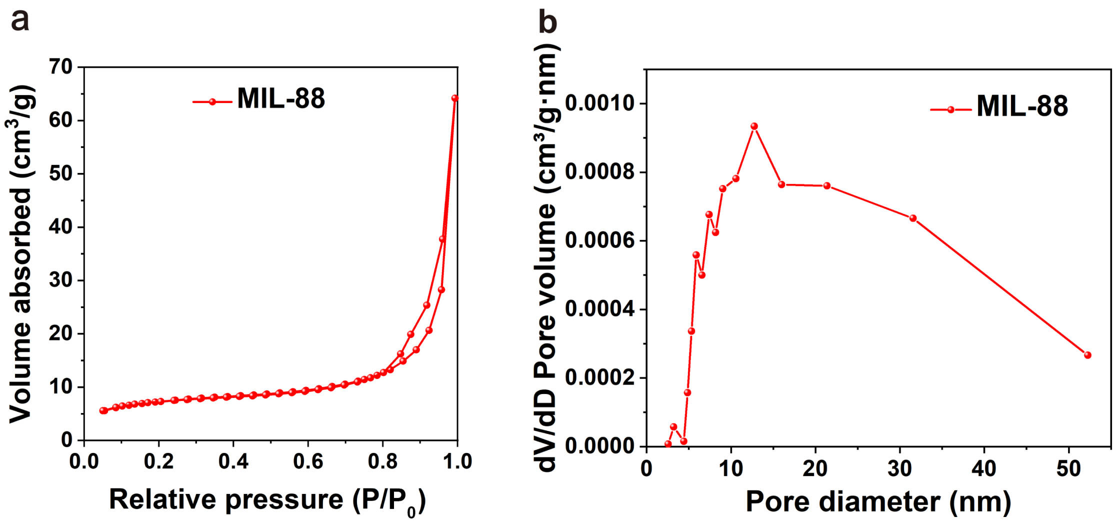


**Figure S3** (a,b) $N_2$ adsorption and desorption isotherms (a) and pore diameter distributions (b) of MIL-88.

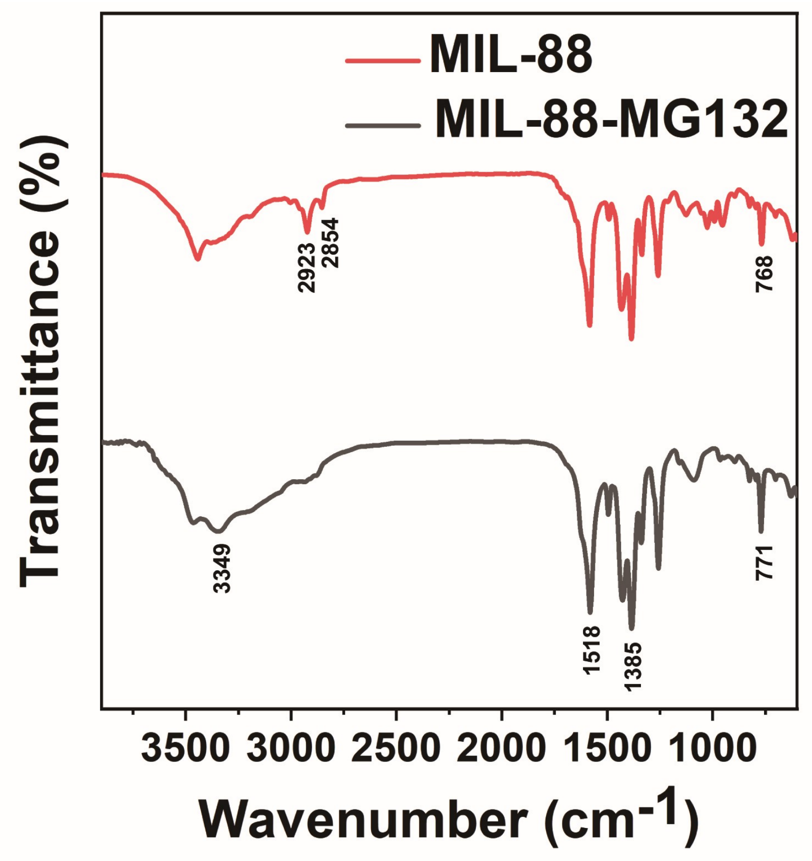

**Figure S4** FTIR spectra of MIL-88 and MIL-88-MG132.

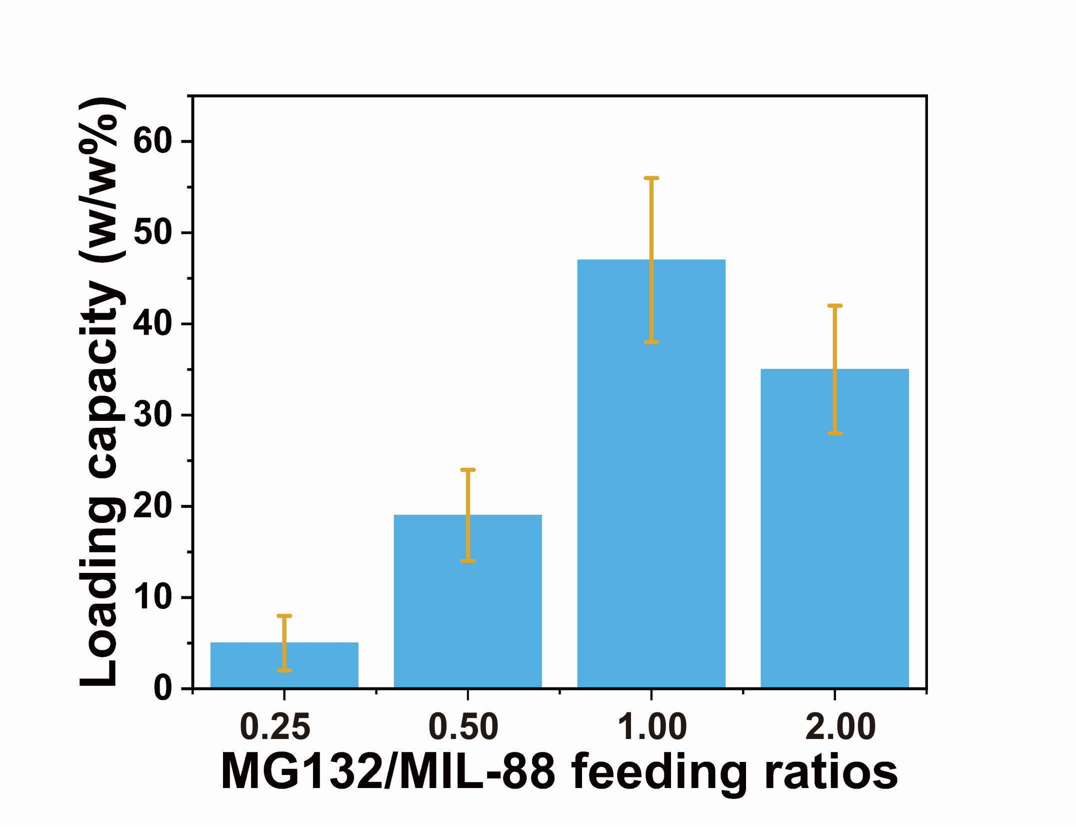


**Figure S5** Loading capacity of MG132 in MIL-88-MG132 as a function of the feeding ratio of MG132/MIL-88.

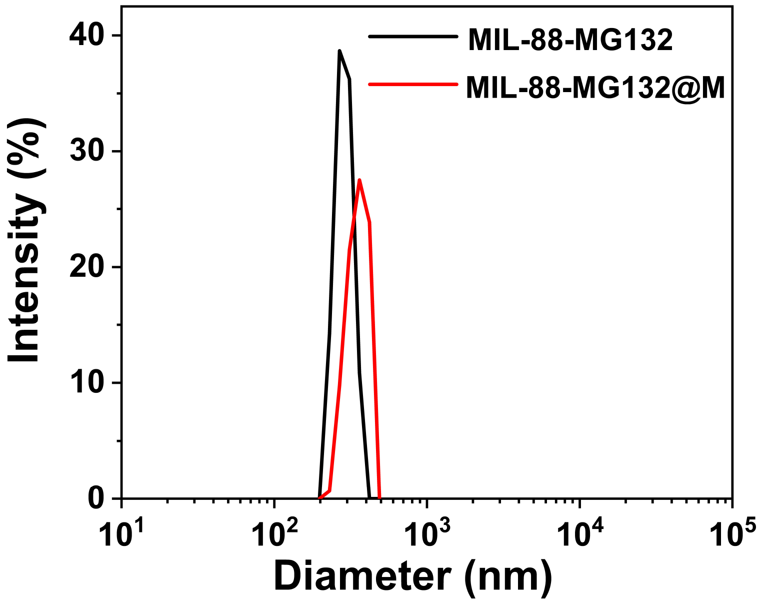


**Figure S6** Hydrodynamic diameters of MIL-88-MG132 and MIL-88-MG132@M nanoparticles in PBS.

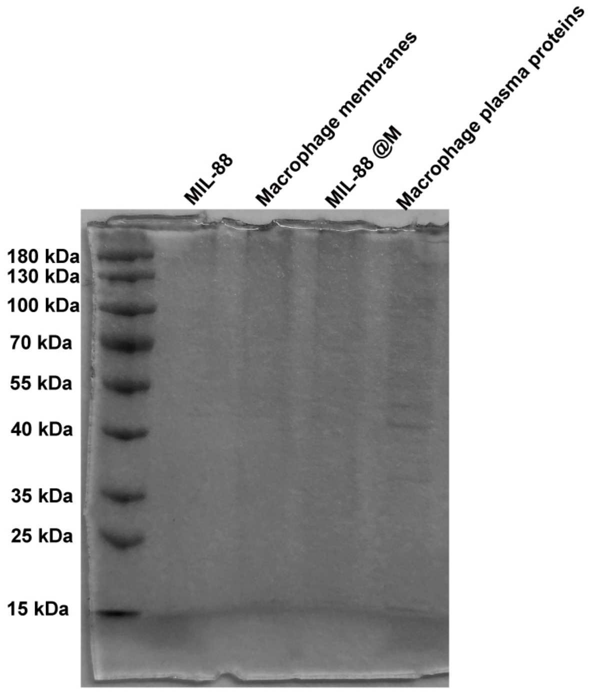


**Figure S7** SDS-PAGE protein identification of MIL-88 and MIL-88@M.

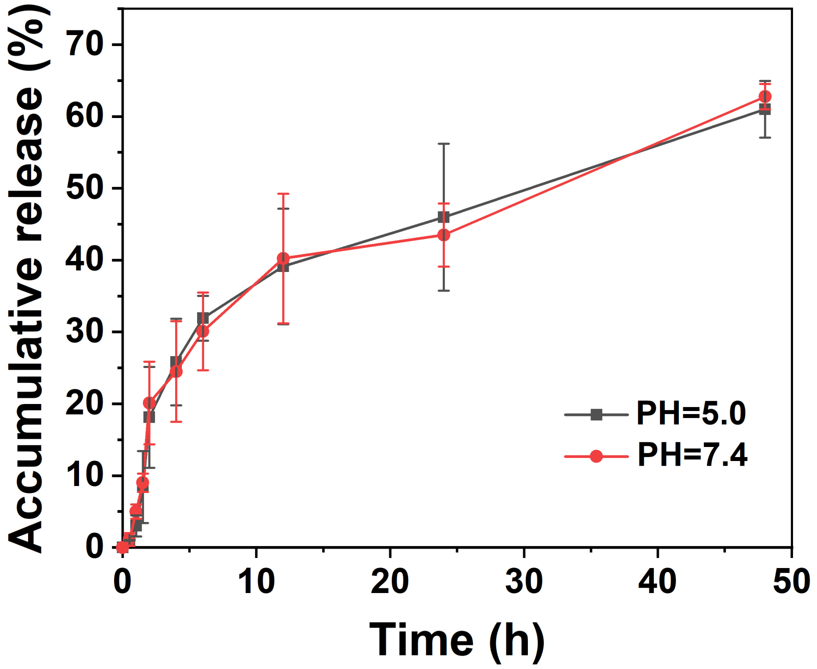


**Figure S8** Release profiles of MG132 from MIL-88-MG132@M in pH = 5.0 or pH = 7.4.

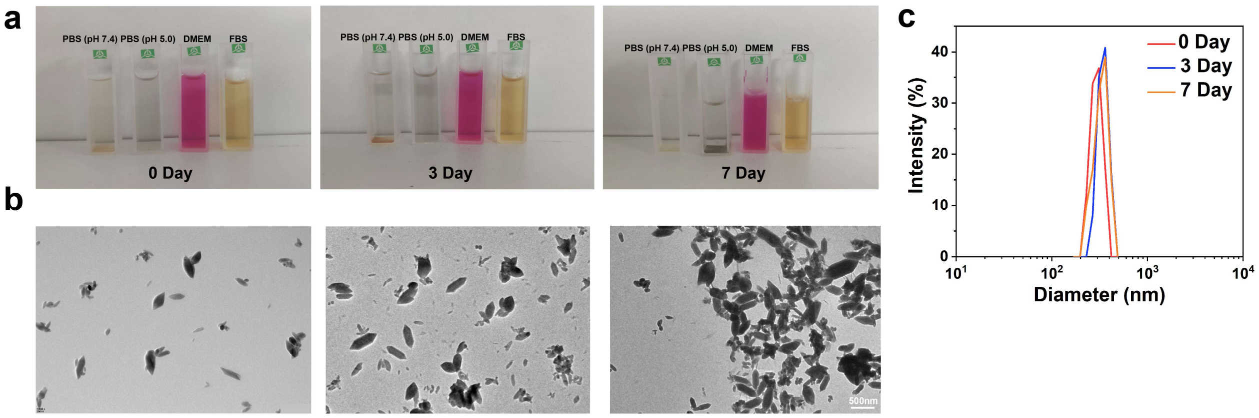


**Figure S9** (a) Digital photos of MIL-88-MG132@M dispersions in different solvents (PBS (pH = 5.0 or = 7.4), dulbecco's modified eagle medium (DMEM), fetal bovine serum (FBS)) for different durations (0 day, 3 day and 7 day). (b,c) TEM images (b) and hydrodynamic diameters (c) of MIL-88-MG132@M dispersion in PBS after different durations (0 day, 3 day and 7 day), respectively.

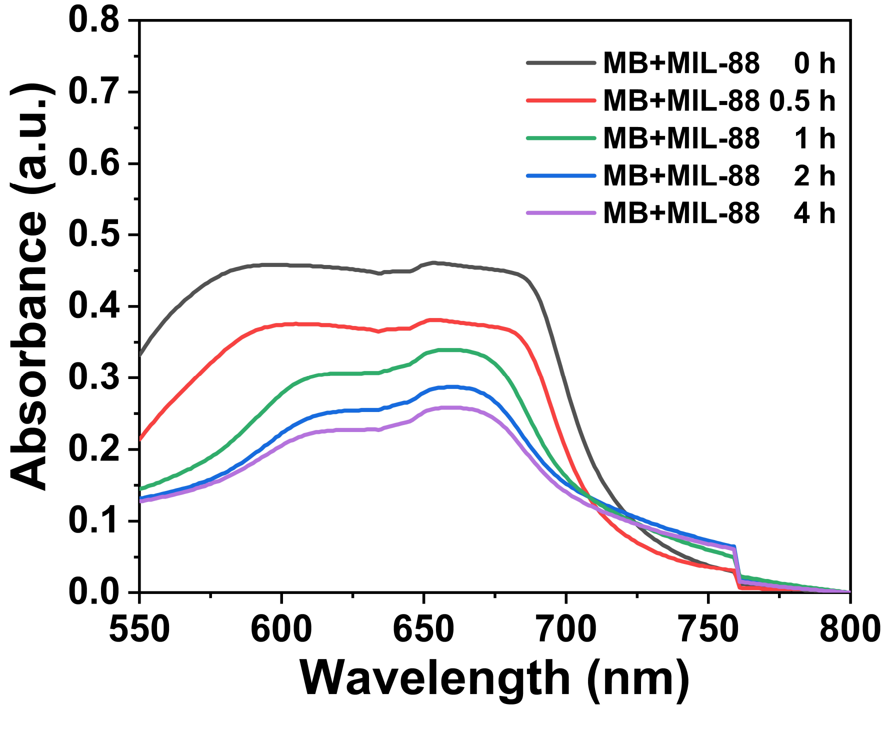


**Figure S10** UV-vis spectra of MB after incubation with MIL-88 for different time durations.

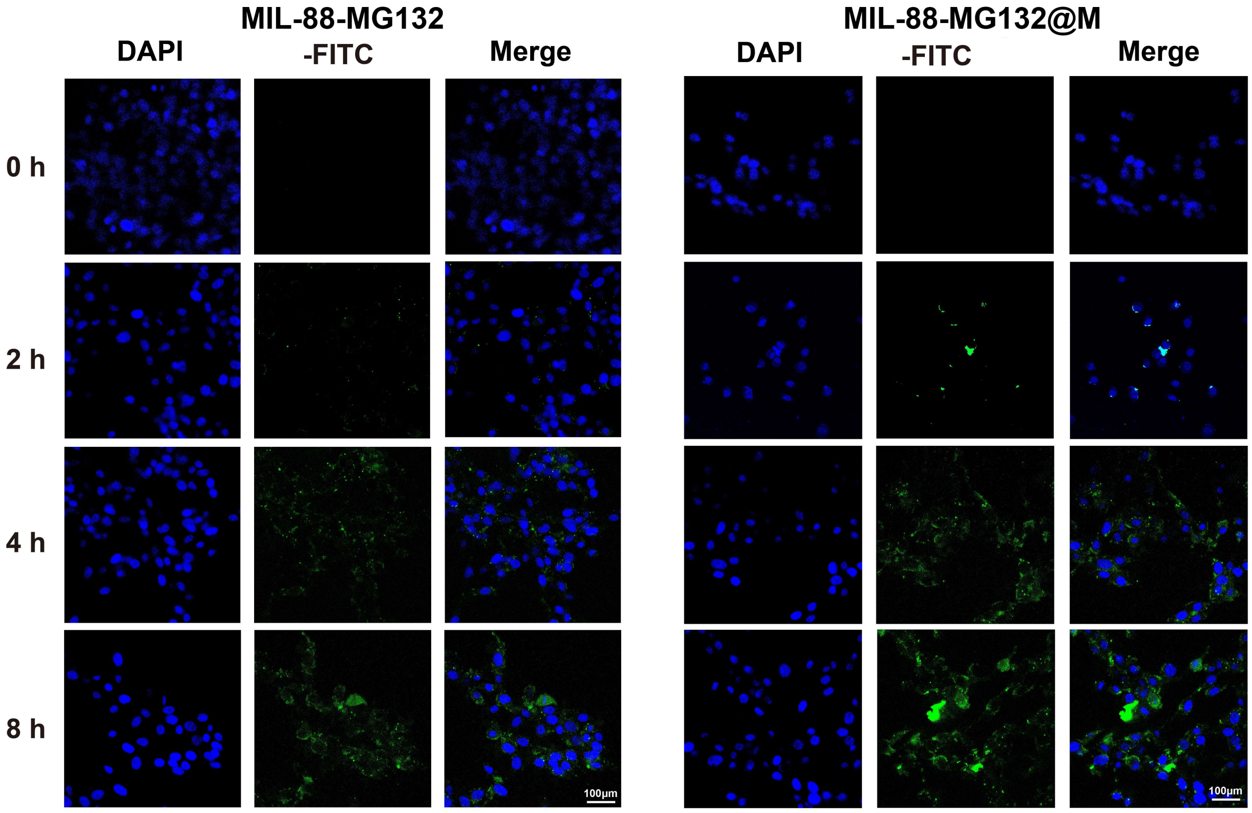


**Figure S11** CLSM images of CT-26 cells after incubation with FITC-labeled MIL-88-MG132 and MIL-88-MG132@M nanoparticles for different time periods (scar bar: 100 μm).

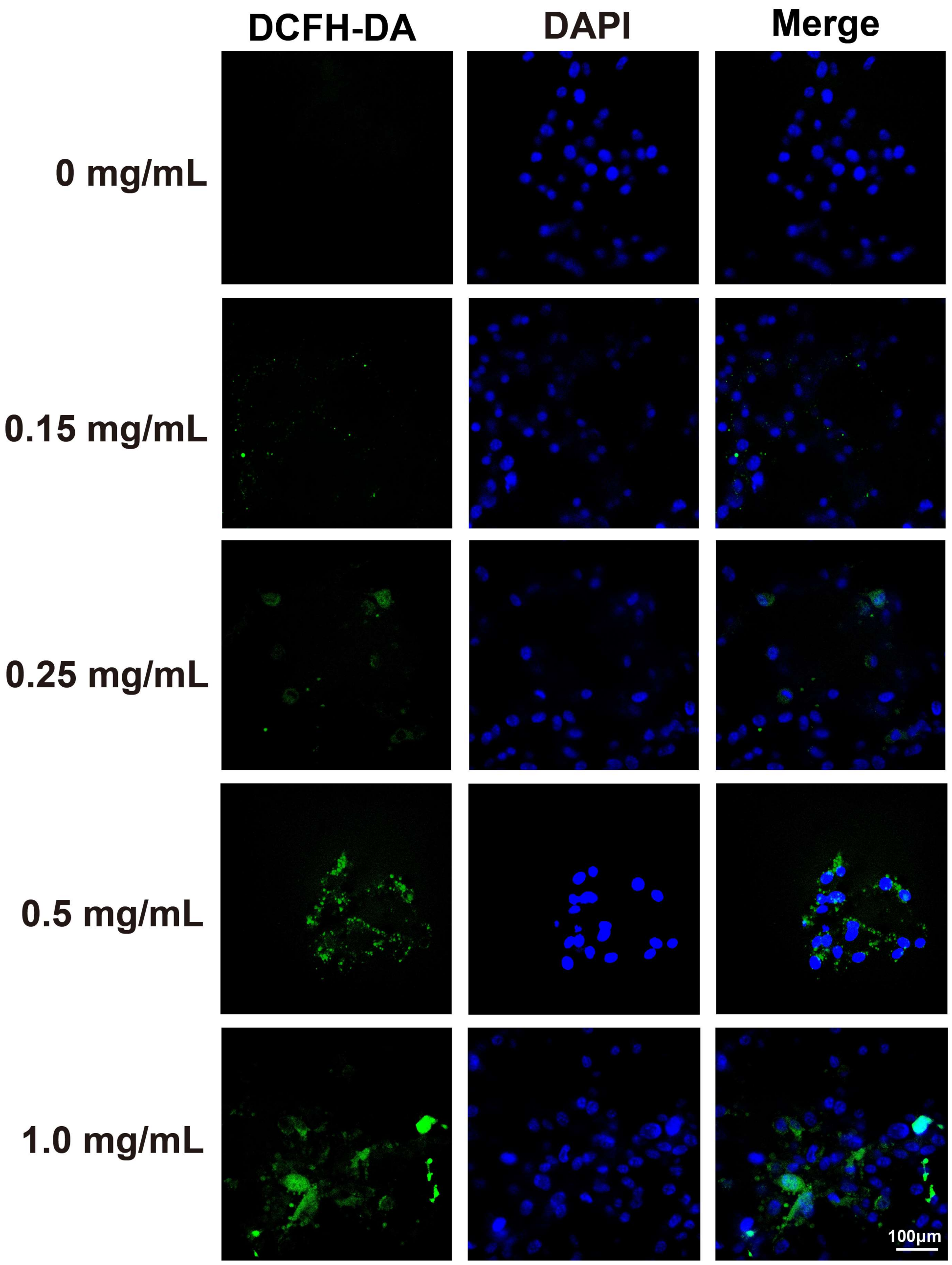


**Figure S12** CLSM images of CT-26 cells after incubation with MIL-88-MG132@M nanoparticles with varied concentrations and subsequent DCFH-DA staining (scar bar: 100 μm).

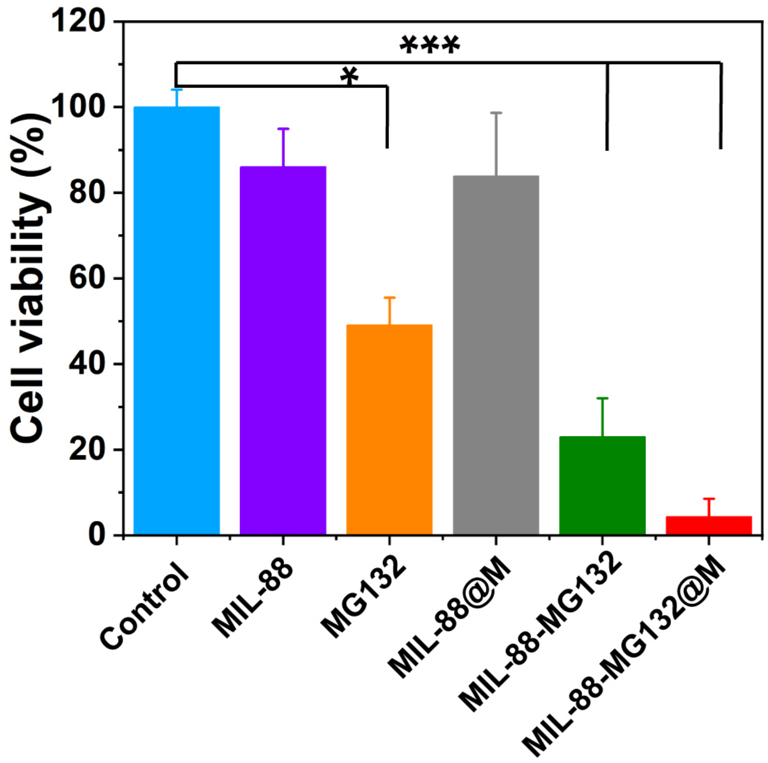


**Figure S13** The viability of CT-26 cells after different treatments in various groups including control, MIL-88, MG132, MIL-88@M, MIL-88-MG132 and MIL-88-MG132@M. Data are expressed as mean±SD (n=5). Statistical significance between all different groups was determined by two-sided Student's t-test, and *P< 0.05 and ***P < 0.001.

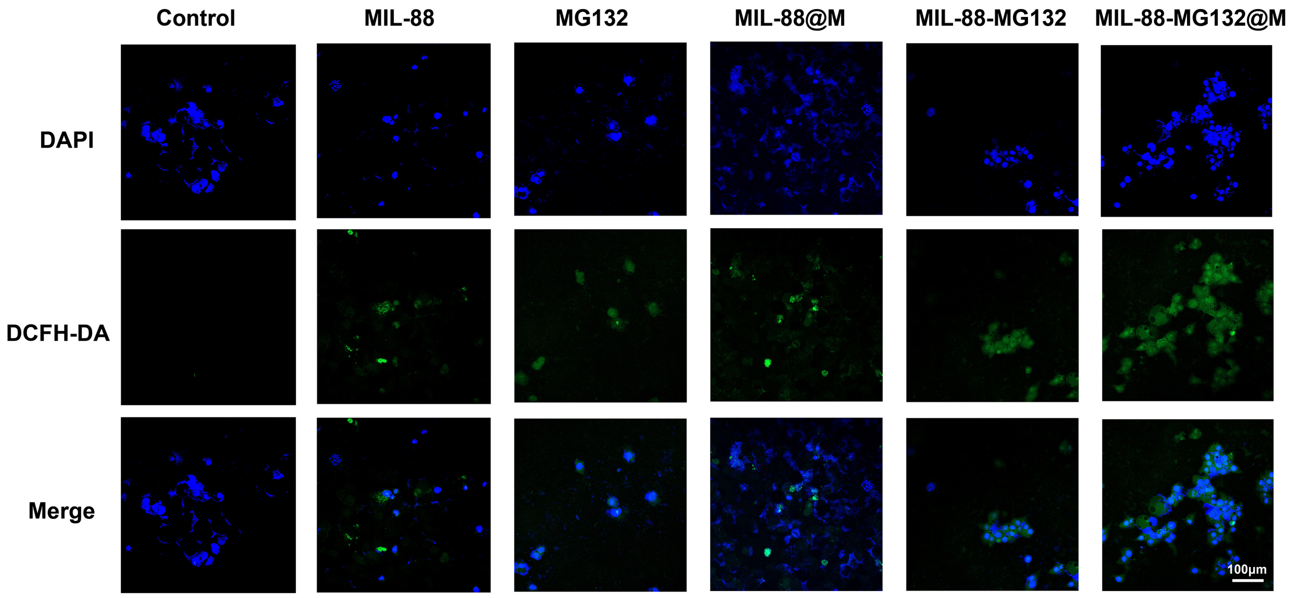


**Figure S14** CLSM images of HCT-116 cells after different corresponding treatments and subsequent DCFH-DA staining.

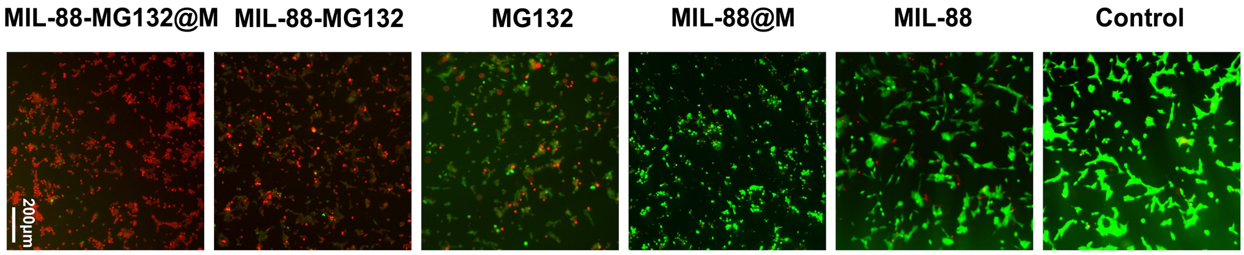


**Figure S15** CLSM images of HCT-116 cells co-stained by calcein AM/PI after different corresponding treatments.

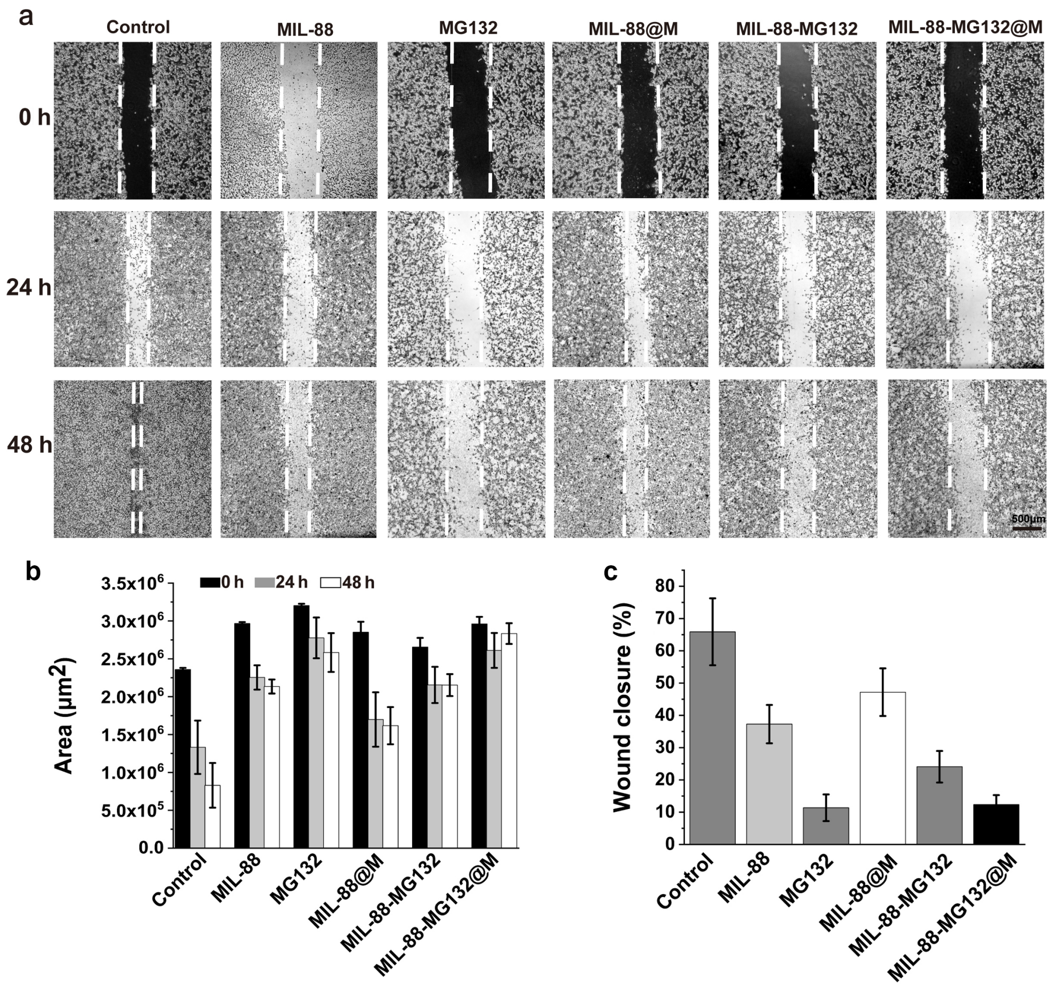


**Figure S16** Cell scratch test of CT-26 cells after different treatments in various groups including control, MIL-88, MG132, MIL-88@M, MIL-88-MG132 and MIL-88-MG132@M: (a) scratch images, (b) scratch area and (c) wound closure. Data are expressed as mean ± SD (n=3).

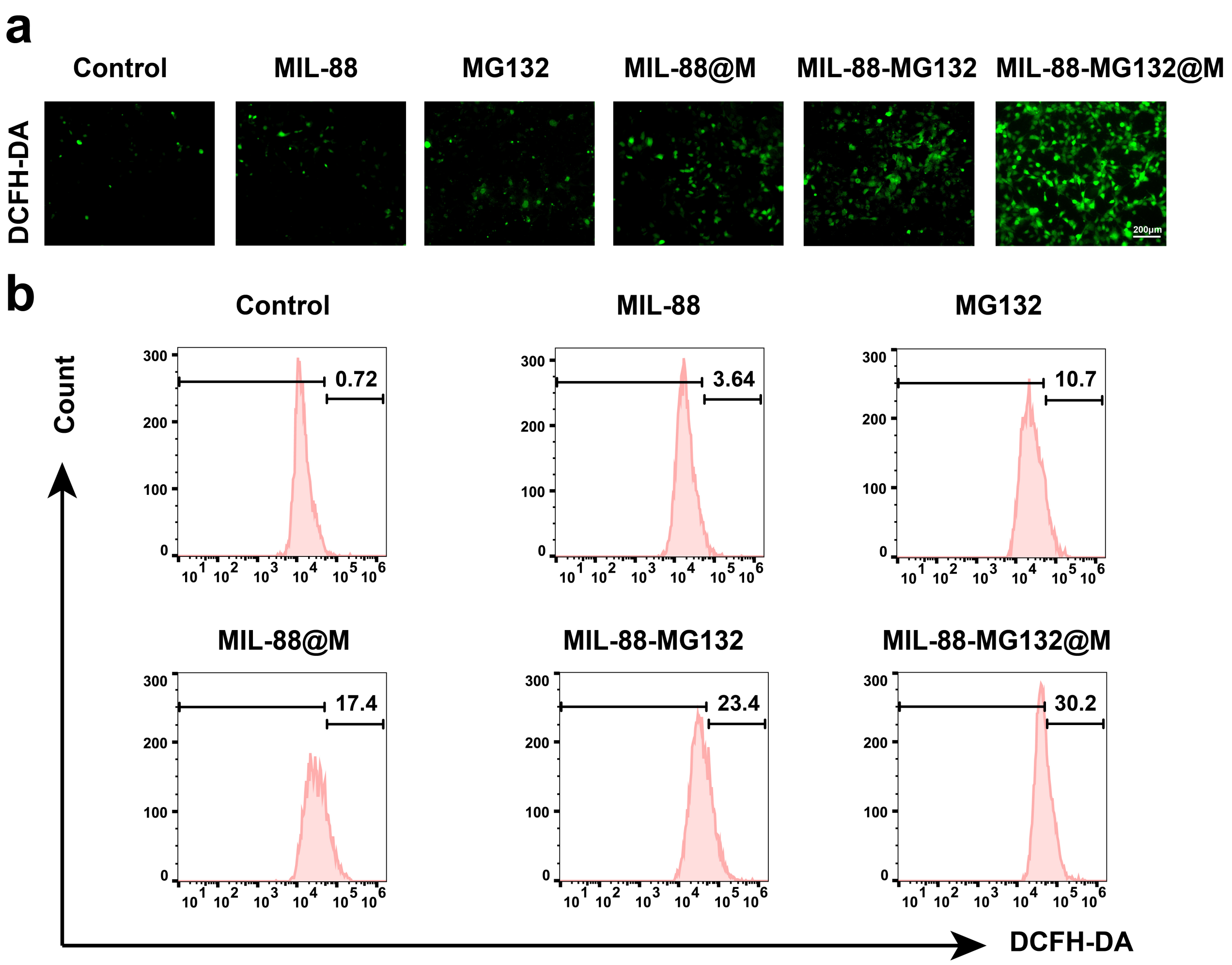


**Figure S17** (a) CLSM images of Hepa 1-6 cells after different corresponding treatments and subsequent DCFH-DA staining. (b) FCM patterns of ROS-indicator-stained Hepa 1-6 cells for tracking ROS levels after different treatments.

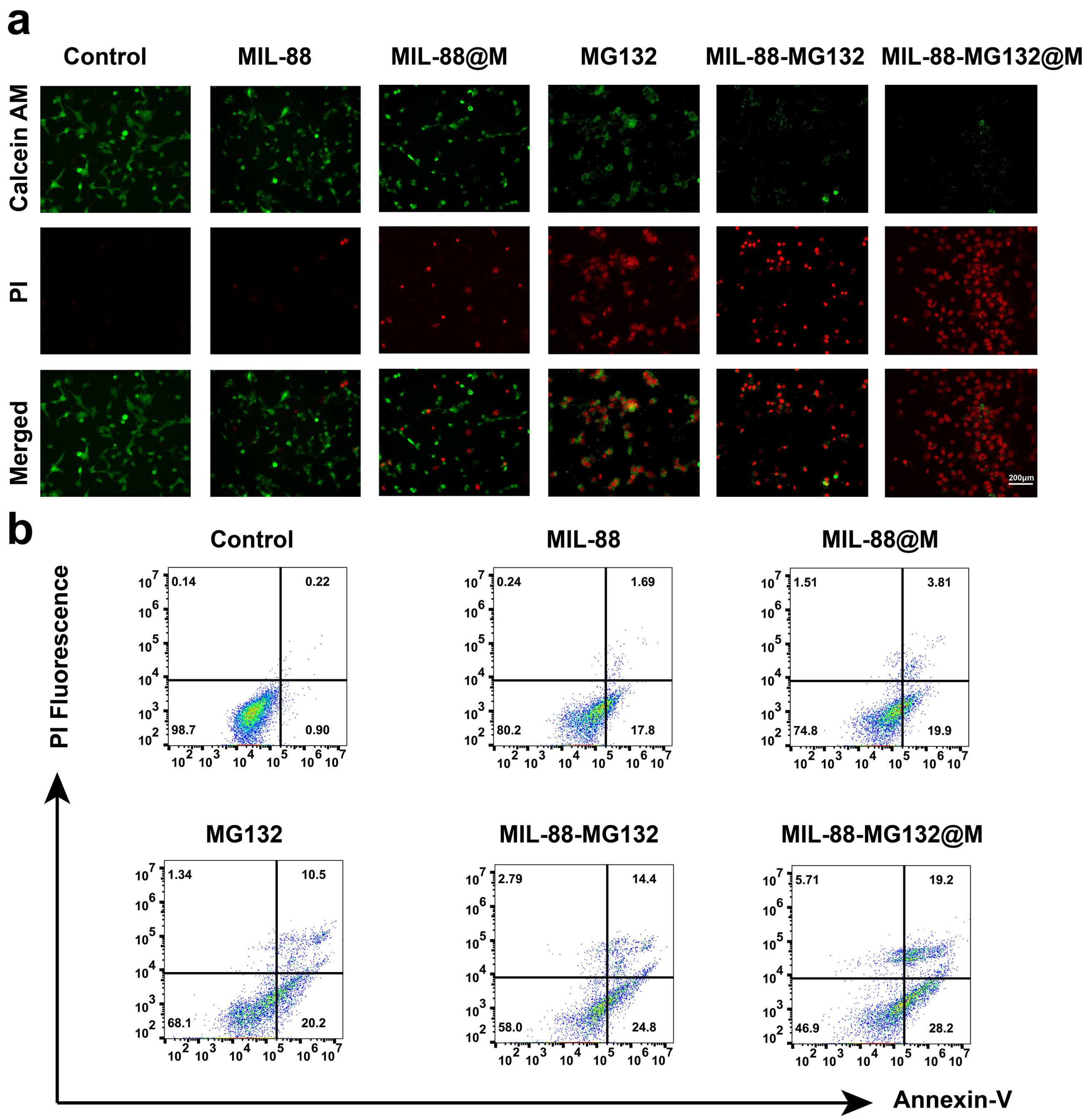


**Figure S18** (a) CLSM images of Hepa 1-6 cells co-stained by calcein AM/PI after different corresponding treatments. (b) FCM patterns of Hepa 1-6 cells co-stained by Annexin-V/PI after different corresponding treatments.

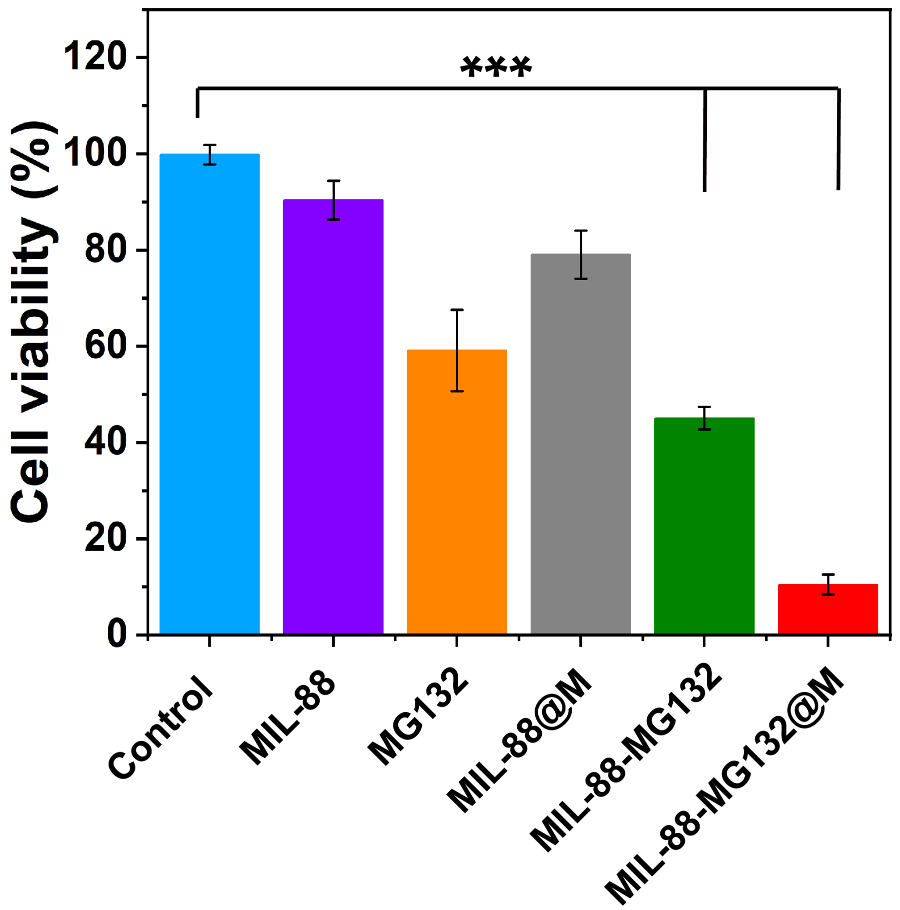


**Figure S19** The viability of Hepa 1-6 cells after different treatments in various groups including control, MIL-88, MG132, MIL-88@M, MIL-88-MG132 and MIL-88-MG132@M. Data are expressed as mean±SD (n=5). Statistical significance between all different groups was determined by two-sided Student's t-test, and ***P < 0.001.

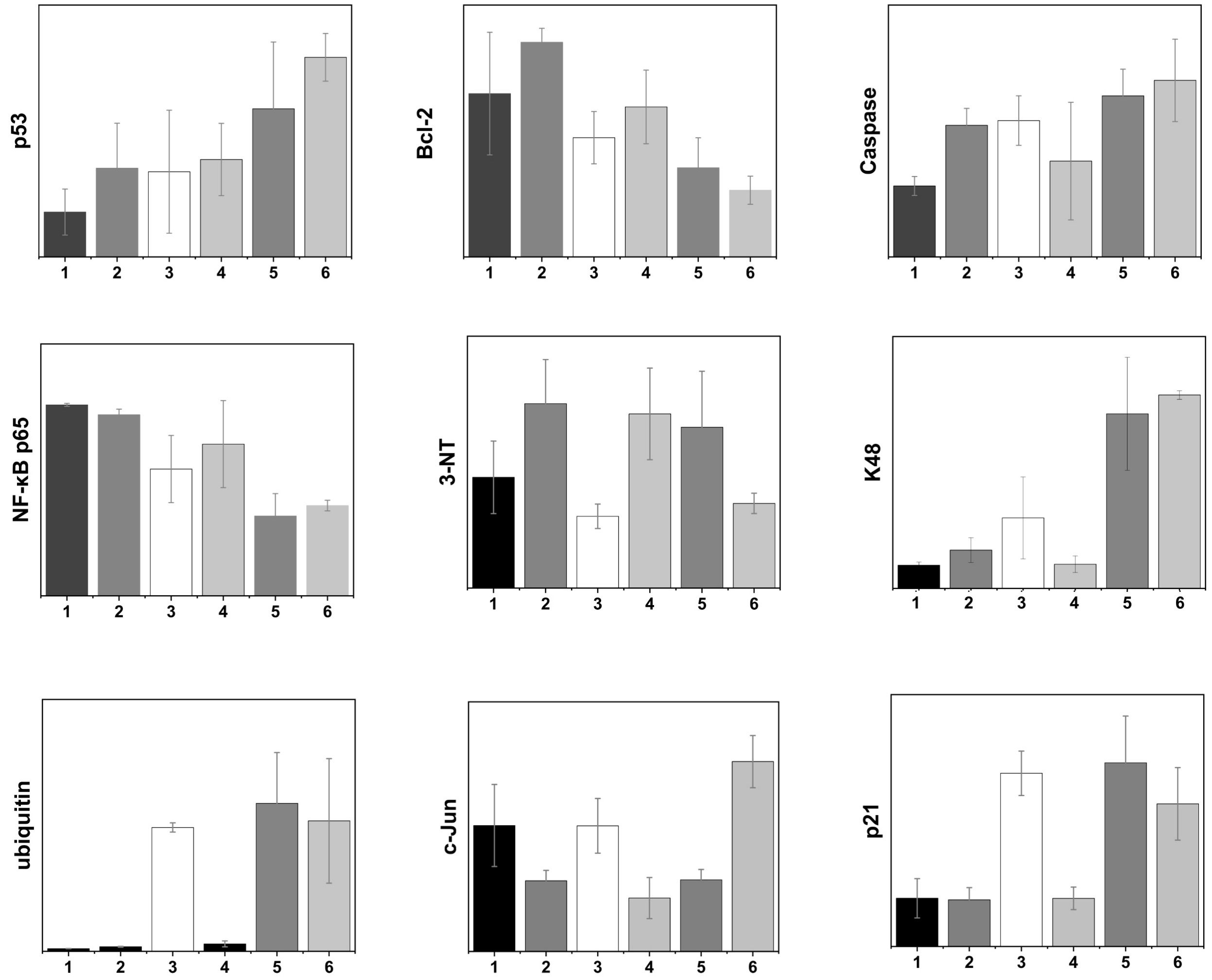


**Figure S20** Relative expression levels of ROS-sensitive proteins (i.e., caspase and 3-NT), apoptosis-related proteins (e.g., p53, Bcl-2, cyclins D1, NF-kB p65), proteasome inhibitors (K48, Ubiquitin) and proteasomal substrate protein (e.g., c-Jun and p21) in CT-26 cells that experienced different treatments in corresponding groups 1-6. Note, groups 1-6 represent control, MIL-88, MG132, MIL-88@M, MIL-88-MG132 and MIL-88-MG132@M, respectively. Data are expressed as mean ± SD (n=3).

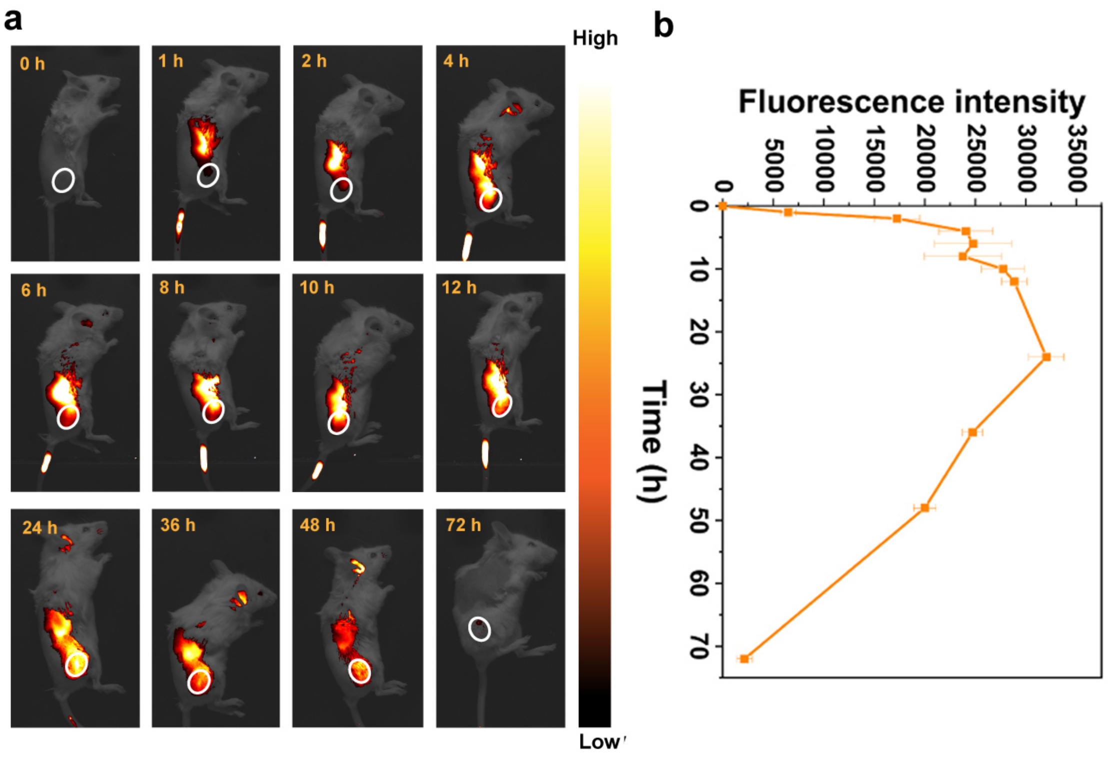


**Figure S21** (a,b) In vivo fluorescence images (a) and the corresponding fluorescence intensity (b) of CT26 tumor-bearing mice after intravenous administration of IR780-labeled MIL-88-MG132 at different time points. Data are expressed as mean ± SD (n=3).

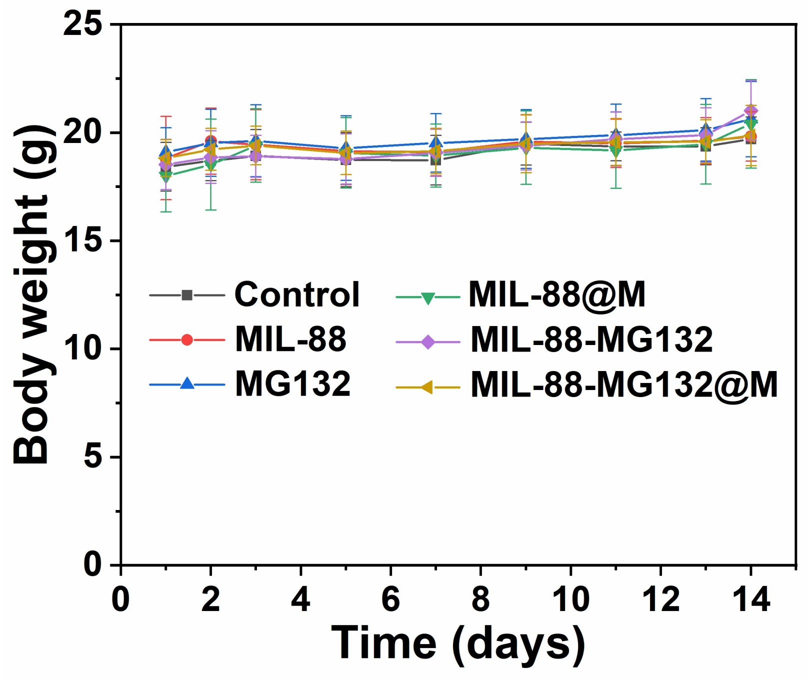


**Figure S22** Time-dependent body weights of CT-26 tumor-bearing mice after different treatments in groups 1-6. Data are expressed as mean ± SD (n=5). Note, MIL-88 dose: 20 mg/kg; and groups 1-6 represent control, MIL-88, MG132, MIL-88@M, MIL-88-MG132 and MIL-88-MG132@M, respectively.

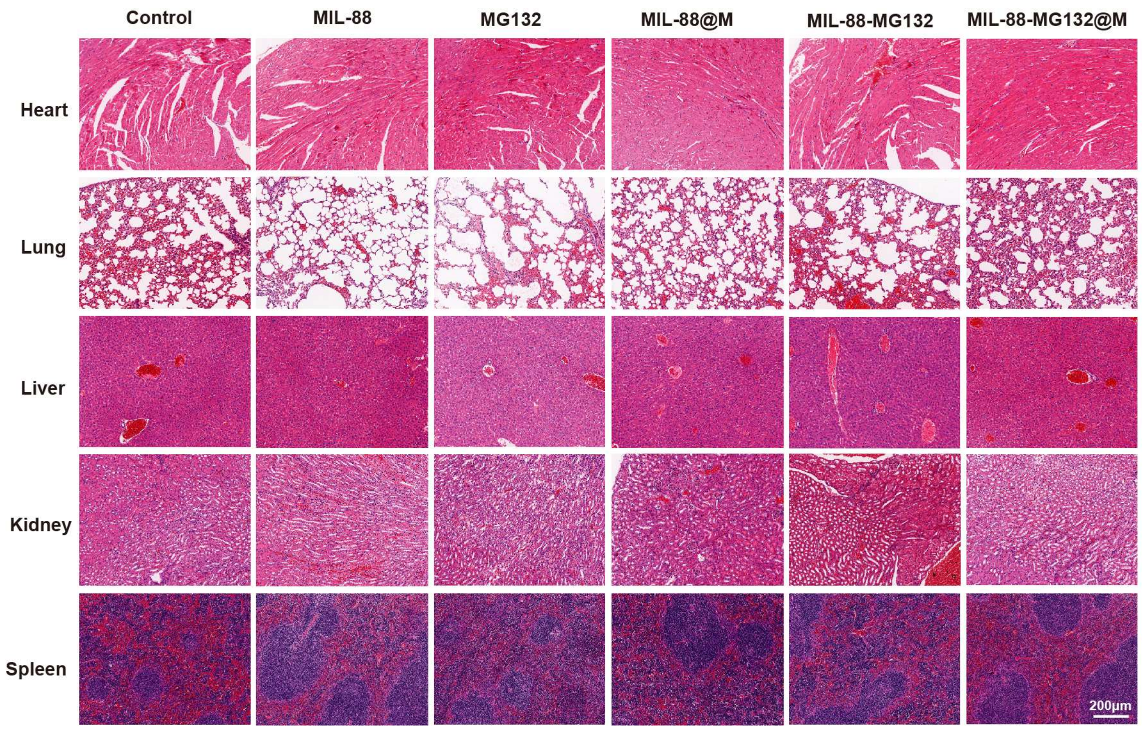


**Figure S23** H&E staining microscopic images of normal organ slices (i.e., heart, liver, spleen, lung and kidney) in CT-26 tumor-bearing mice that experienced different treatments in groups 1-6. Note, MIL-88 dose: 20 mg/kg; and groups 1-6 represent control, MIL-88, MG132, MIL-88@M, MIL-88-MG132 and MIL-88-MG132@M, respectively.

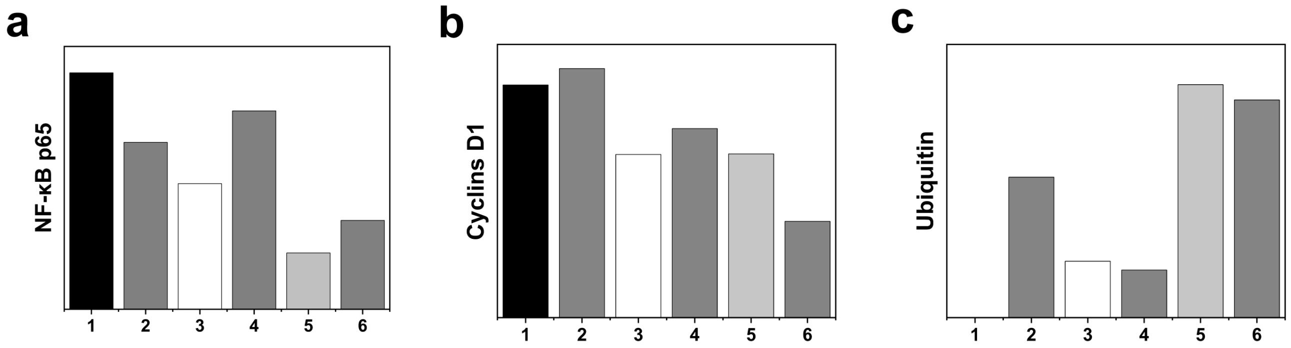


**Figure S24** (a-c) Relative expression levels of various proteins including NF-κB p65, Cyclins D1 and Ubiquitin in CT-26 tumors harvested from CT-26 tumor-bearing mice that experienced different treatments in groups 1-6. Note, MIL-88 dose: 20 mg/kg; and groups 1-6 represent control, MIL-88, MG132, MIL-88@M, MIL-88-MG132 and MIL-88-MG132@M, respectively.

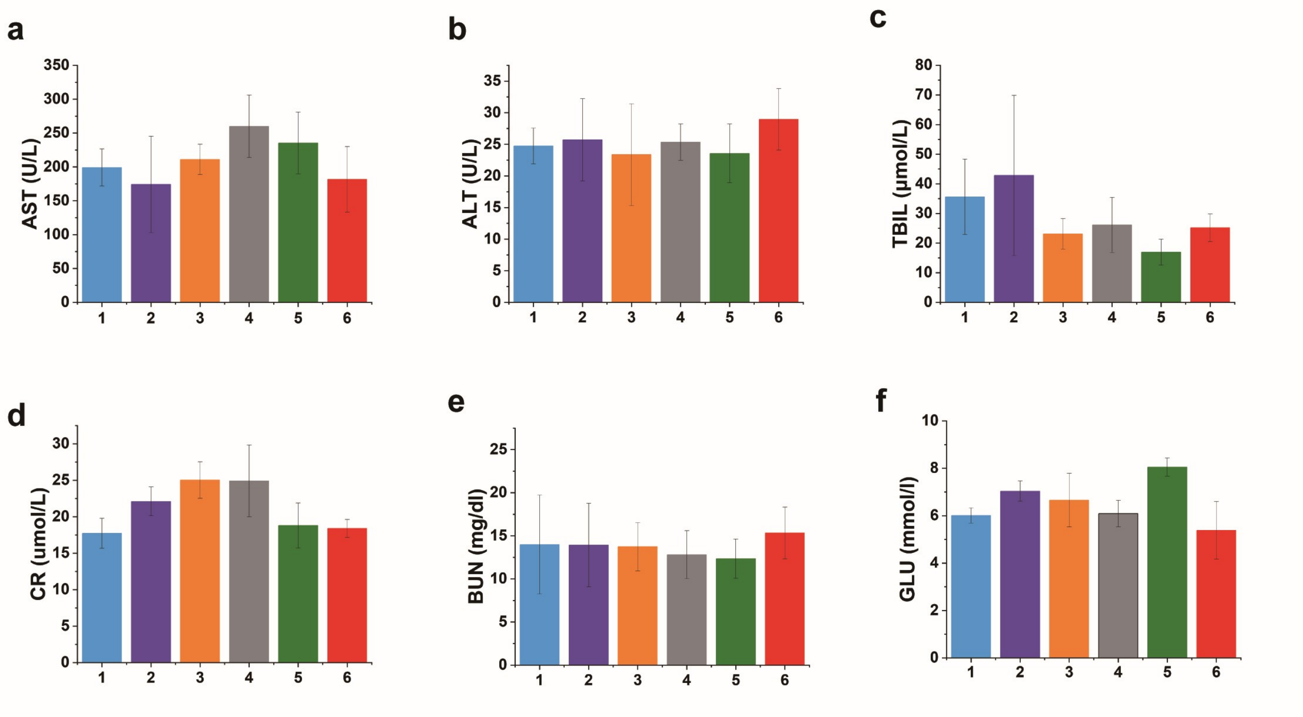


**Figure S25** (a-f) Liver and renal biochemical indices including aspartate aminotransferase (AST) (a), alanine aminotransferase (ALT) (b), total bilirubin (TBIL) (c), creatinine (CR) (d), blood urea nitrogen (BUN) (e) and glucose (GLU) (f), and the blood samples were collected from Balb/c nude mice bearing CT-26 tumors after 21 days post-different treatments in different groups. Groups 1-6 represent control, MIL-88, MG132, MIL-88@M, MIL-88-MG132, MIL-88-MG132@M, respectively. Data are expressed as mean ± SD (n=5).

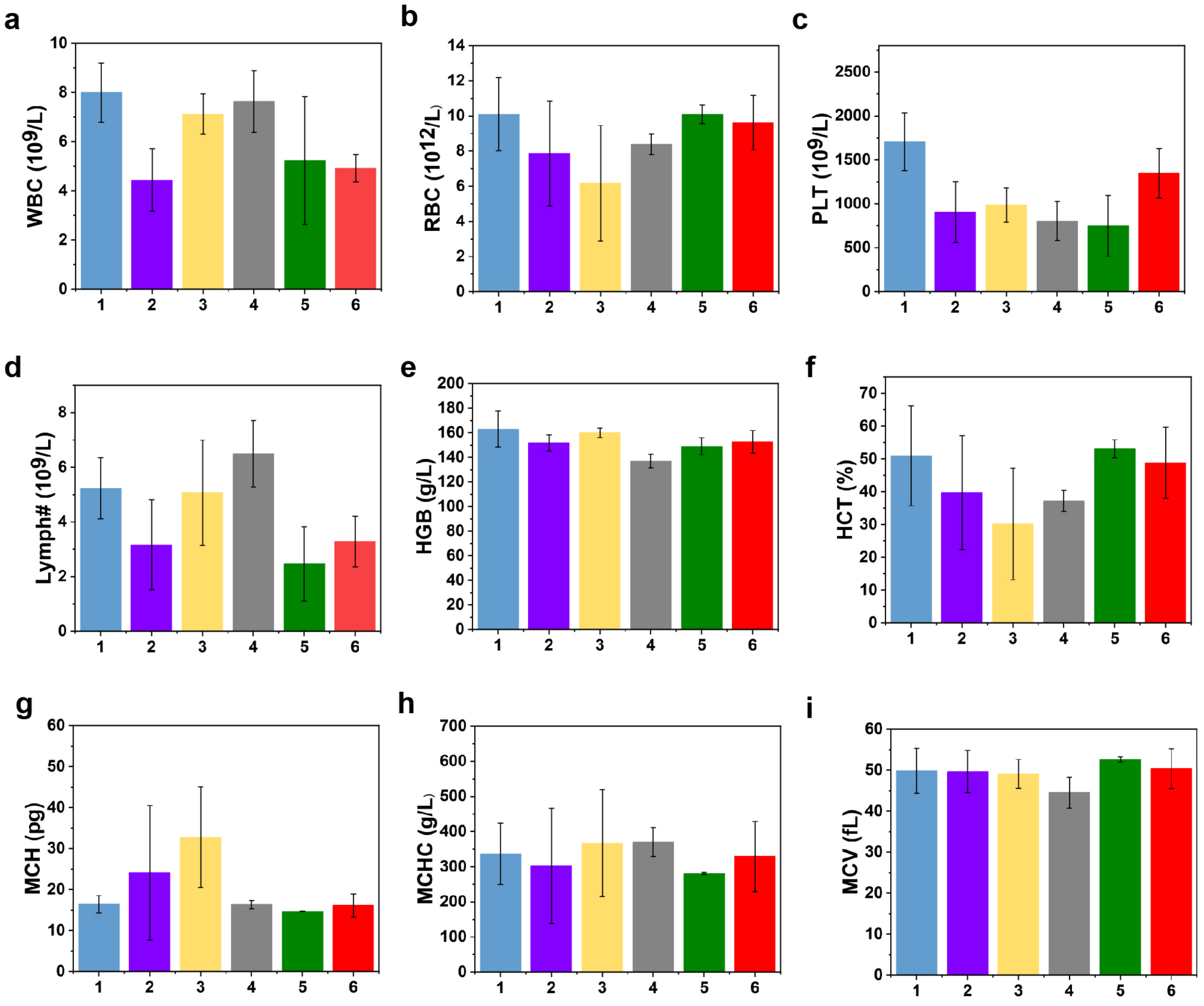


**Figure S26** (a-i) Routine blood parameters including white blood cell (WBC) (a), red blood cell (RBC) (b), platelet (PLT)(c), lymphocyte (LYM) (d), hemoglobin (HGB) (e), hematocrit (HCT) (f), mean corpuscular hemoglobin (MCH) (g), and mean corpuscular hemoglobin concentration (MCHC) (h) and mean corpuscular volume (MCV) (i), and the blood samples were collected from Balb/c nude mice bearing CT-26 tumors after 21 days post-different treatments in different groups. Groups 1-6 represent control, MIL-88, MG132, MIL-88@M, MIL-88-MG132, MIL-88-MG132@M, respectively. Data are expressed as mean ± SD (n=5).

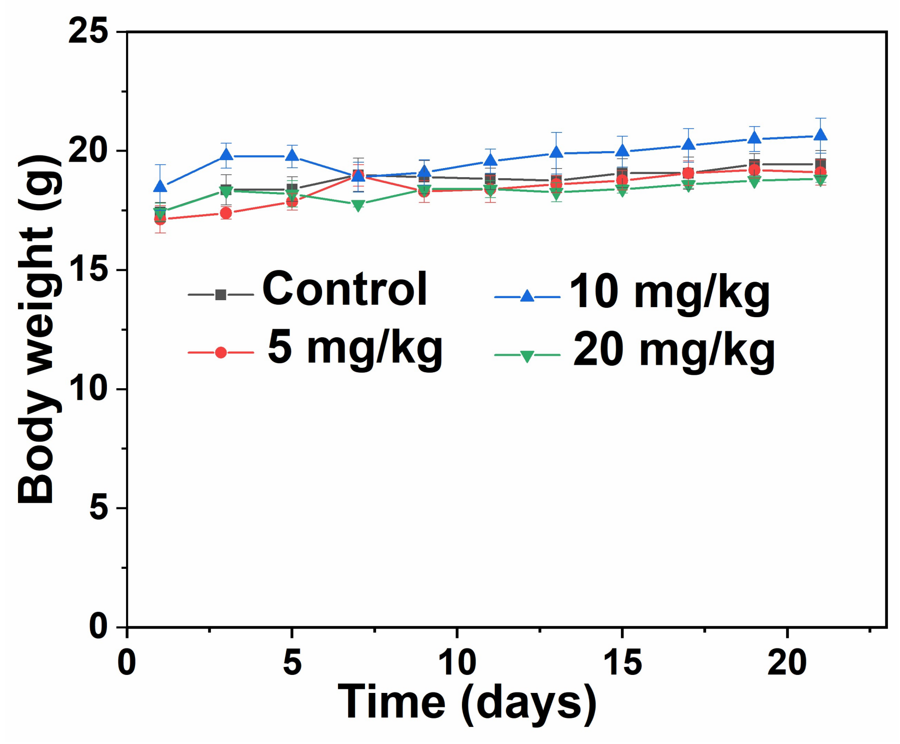


**Figure S27** Time-dependent body weight profiles of Balb/c mice after intravenous injection of MIL-88-MG132@M with different doses (5 mg/kg, 10 mg/kg and 20 mg/kg) and PBS (control) during 21-day observation period. Data are expressed as mean ± SD (n=5).

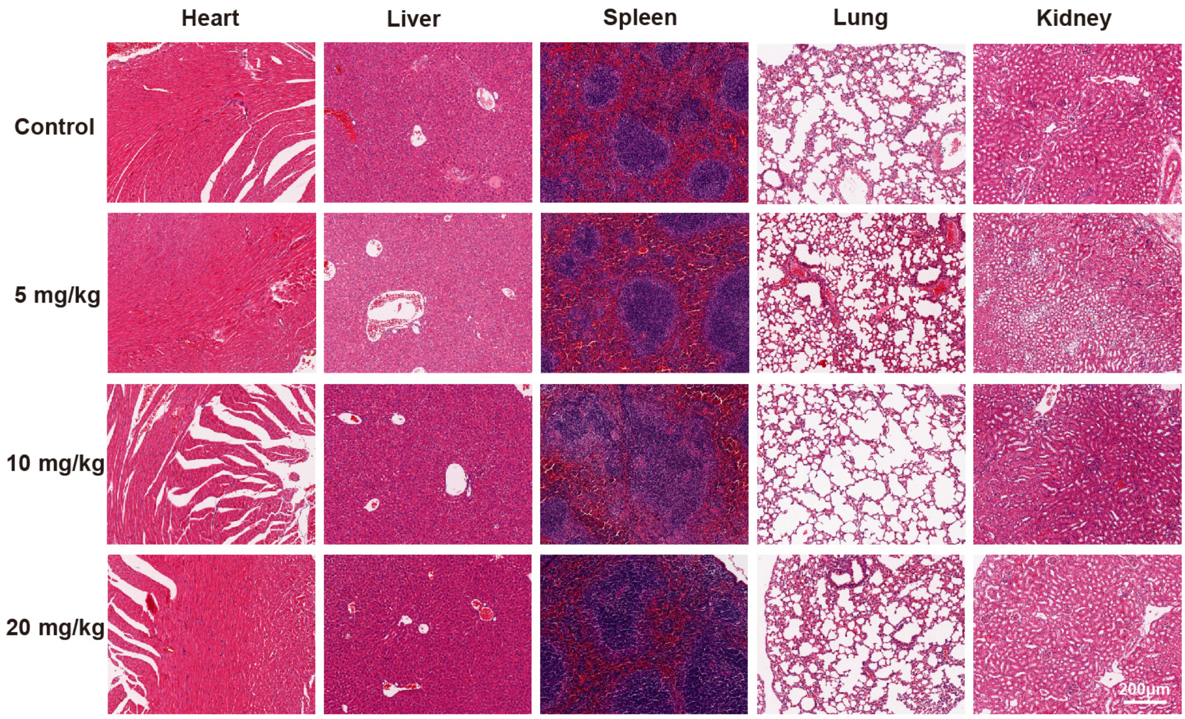


**Figure S28** H&E-stained images of normal organs (i.e., heart, liver, spleen, lung and kidney) in mice that experienced caudal vein injection of MIL-88-MG132@M with varied concentrations (5 mg/kg, 10 mg/kg, 20 mg/kg) and another 21 days feeding. Scale bar: 200 μm.

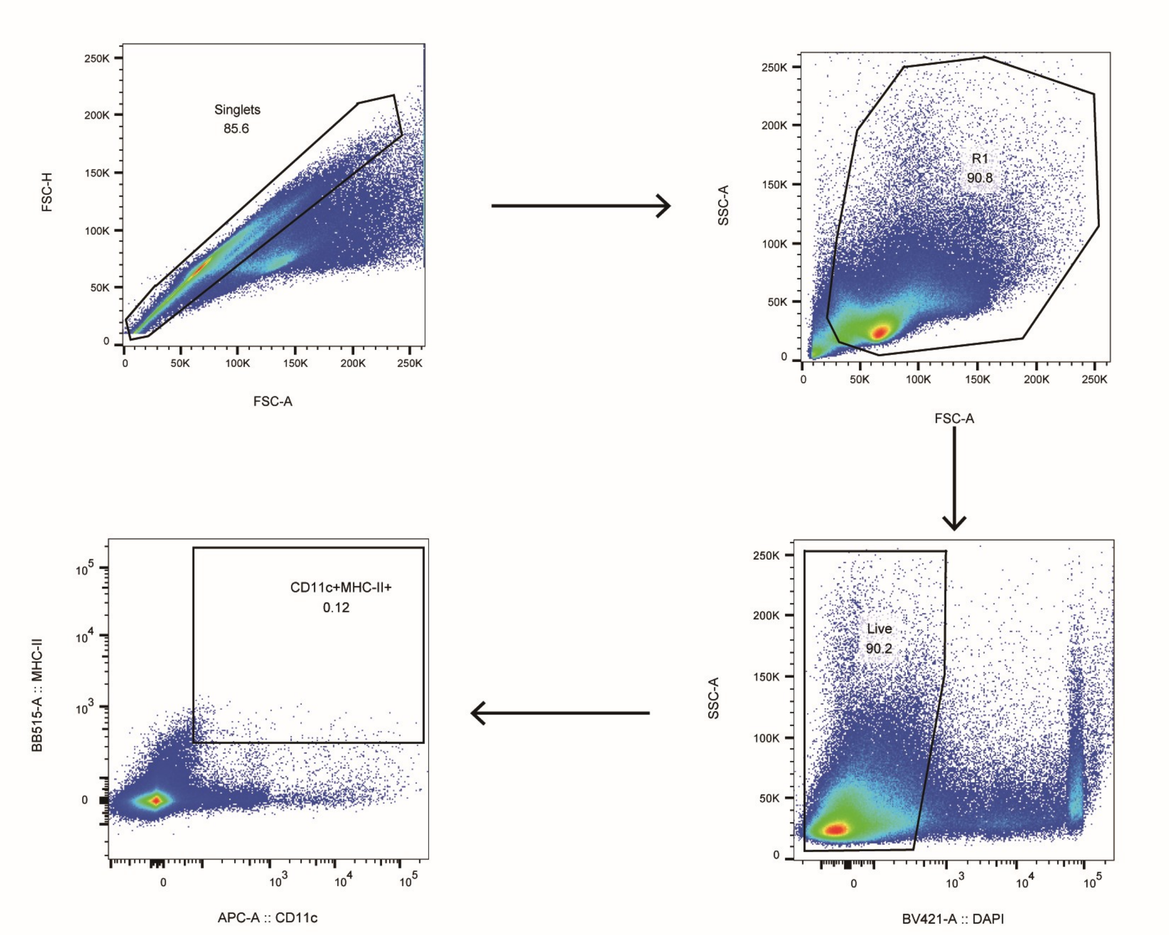


**Figure S29** Representative example of scatter lots from cytometry analyses based on 2- color showing the DCs from a mice spleen. Arrows indicate the gating strategy.
